# Functionalized hybrid magnetic catalytic systems on micro- and nanoscale utilized in organic synthesis and degradation of dyes

**DOI:** 10.1039/d1na00818h

**Published:** 2022-02-09

**Authors:** Fatemeh Ganjali, Amir Kashtiaray, Simindokht Zarei-Shokat, Reza Taheri-Ledari, Ali Maleki

**Affiliations:** Catalysts and Organic Synthesis Research Laboratory, Department of Chemistry, Iran University of Science and Technology Tehran 16846-13114 Iran r_taheri94@chem.iust.ac.i maleki@iust.ac.ir +98-21-73021584 +98-21-73228313

## Abstract

Herein, a concise review of the latest developments in catalytic processes involving organic reactions is presented, focusing on magnetic catalytic systems (MCSs). In recent years, various micro- and nanoscale magnetic catalysts have been prepared through different methods based on optimized reaction conditions and utilized in complex organic synthesis or degradation reactions of pharmaceutical compounds. These biodegradable, biocompatible and eco-benign MCSs have achieved the principles of green chemistry, and thus their usage is highly advocated. In addition, MCSs can shorten the reaction time, effectively accelerate reactions, and significantly upgrade both pharmaceutical synthesis and degradation mechanisms by preventing unwanted side reactions. Moreover, the other significant benefits of MCSs include their convenient magnetic separation, high stability and reusability, inexpensive raw materials, facile preparation routes, and surface functionalization. In this review, our aim is to present at the recent improvements in the structure of versatile MCSs and their characteristics, *i.e.*, magnetization, recyclability, structural stability, turnover number (TON), and turnover frequency (TOF). Concisely, different hybrid and multifunctional MCSs are discussed. Additionally, the applications of MCSs for the synthesis of different pharmaceutical ingredients and degradation of organic wastewater contaminants such as toxic dyes and drugs are demonstrated.

## Abstract 2

A comprehensive survey is presented to review the recent advances of functionalized hybrid magnetic catalytic systems for organic reactions and degradation of dyes.

## Graphical abstract

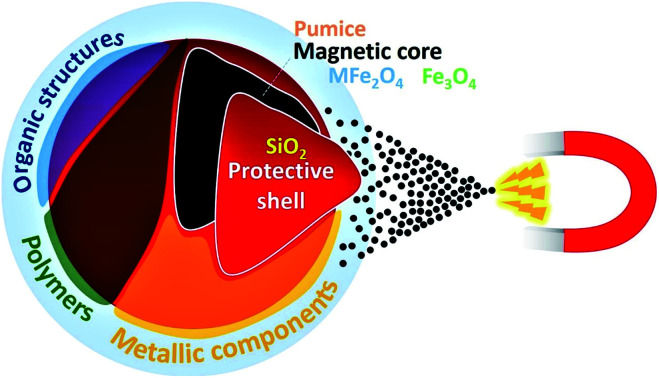

## 1. Introduction

The synthesis of pharmaceutical compounds as the foundation of the pharmaceutical industry conventionally requires complex synthetic routes. However, this problem can be alleviated by using appropriate catalysts in the synthetic process.^1–4^ Also, the discharge of dangerous and toxic drugs in water, which has far-reaching effects on the aquatic ecosystem and the health of living creatures, has become a major problem. One solution to this issue is to catalytically degrade drugs into low-risk components. However, separating homogeneous catalysts from the reaction medium is difficult, which is why recent research has focused on the use of heterogeneous catalytic systems.^5^ Heterogeneous catalytic systems render the catalyst and products in separable phases to speed up the effortless separation and lessen the reaction time with a higher yield.^6^

Generally, among the extensive and diverse collection of heterogeneous catalytic systems, magnetic catalytic systems (MCSs) have attracted increasing attention from scientists due to their substantial benefits.^7–9^ Also, nanomaterial-based catalysts are highly regarded owing to their high aspect ratio.^10–12^ Initially, magnetic catalysts facilitate the purification and workup process.^13^ As reported, for the removal of a magnetic catalyst, a simple process is to hold an external magnet under the reaction flask and decant the medium, where a magnetic field of 20 emu††Electromagnetic unit. g^−1^ or higher is required.^14^ Furthermore, in the case of utilizing nanoscale magnetic catalysts, high reaction yields are obtained using a small amount of nanocatalyst due to their high aspect ratio. These heterogeneous catalysts are affordable and satisfactory species for industrial applications.^15^

Another reason to employ heterogeneous MCSs is their facile separation. Heterogeneous MCSs retain their stability even after alternating recycling runs, as long as the structural stability of the catalyst is maintained. Indeed, metallic-based heterogeneous MCSs such as iron demonstrate no significant decline in catalytic performance over several sequential recycling procedures, where thermogravimetric analysis can confirm these claims.^16^ In addition, many studies have been devoted to functionalizing MCSs or combining them with other substances to enhance the functions and applications of catalytic systems.^17–21^ Reportedly, a heterogeneous MCS, *i.e.*, amine-functionalized Fe_3_O_4_@SiO_2_ nanoparticles grafted with gallic acid (GA), (Fe_3_O_4_@SiO_2_–NH_2_–GA), presented not only a high yield of α-aminonitriles *via* one-pot synthesis but also exhibited enhanced reusability and structural stability.^22^

Considering that most MCSs are composed of inorganic or natural components,^23^ they are environmentally benign and considered in the green chemistry spectrum.^24^ The most renowned and forerunner species of MCSs is highly magnetic iron oxide nanoparticles (Fe_3_O_4_ NPs) synthesized in an alkaline environment, *i.e.*, pH > 11, *via* a facile co-precipitation route from Fe^2+^ and Fe^3+^ ions. The resulting Fe_3_O_4_ NPs can be modified with an SiO_2_ layer to inhibit the further oxidation of Fe_3_O_4_ to the less magnetic Fe_2_O_3_ species. The silica-coated Fe_3_O_4_ NPs have several surface hydroxyl groups as active sites, which can bind to other species through covalent bonding.^25,26^

Ferrites are another class that can form MCSs. Various ferrites such as zinc ferrite (ZnFe_2_O_4_), nickel ferrite (NiFe_2_O_4_), and cobalt ferrite (CoFe_2_O_4_), with different dopants, can be employed in the preparation of MCS. These materials exhibit different properties, that is, Ni ions in NiFe_2_O_4_ are easily exposed to reduction compared with the Fe ions.^27^ ZnFe_2_O_4_ has a narrow bandgap, moderate photocatalytic activity, and chemical and thermal stability.^28^ CoFe_2_O_4_ has average saturation magnetization, strong anisotropy, and excellent mechanical and chemical stabilities.^29^ Together with these inorganic systems, well-suited, lightweight, and innately magnetic species such as pumice‡‡An igneous rock. are utilized in MCSs. Another advantage of pumice is its highly porous structure, which is formed when gaseous species are emitted from it.^30,31^

These moderate MCSs have caused pharmaceutical synthesis to proceed in aqueous media, which was previously done in organic solvents. Safari *et al.* designed Fe_3_O_4_ located in the space between the lamellae and exterior surface of the montmorillonite (MMT) support, namely, MMT@Fe_3_O_4_, as an eco-friendly catalyst for the synthesis of indeno[1,2-*b*]indolone in aqueous media with gentle conditions, high yield, and convenient recovery.^32^ Further, in another report, Pd NPs were immobilized on melamine-functionalized magnetic chitosan beads (Fe_3_O_4_/CS–Me@Pd) to catalytically reduce *p*-nitrophenol and to conduct Suzuki reaction in aqueous medium.^33^

In this study, melamine played a particularly important role, which not only acts as a platform for the excellent surface distribution of Pd(ii) but also strongly interacts with Pd(ii) to minimize the leaching of the metal NPs. Another notable advantage of this work was its high catalytic activity, as authenticated by the high TON and TOF under mild conditions. Similarly, MCSs are vital for the degradation of pharmaceuticals with high yield in aqueous medium. As a remarkable example, Kargae *et al.* synthesized a ZnFe_2_O_4_@CMC nanobiocomposite to remove ciprofloxacin (CIP), an antibiotic of the fluoroquinolones group, with good removal efficiency (87%) in synthetic samples without introducing any extra oxidizing agents. This nanophotocatalyst followed the “green chemistry” protocols and showed good chemical stability after five times reuse.^34,35^ Also, Taghavi *et al.* introduced the MCS FeNi_3_/SiO_2_/CuS for the photocatalytic degradation of tetracycline under simulated solar light.^36^ Facile recyclability and advanced photocatalytic decomposition, which degrades complex cyclic compounds into simple linear ingredients, and ultimately turn them into CO_2_ and H_2_O, are two remarkable points in their report.

Magnetic biochar (MBC) composites are members of MCSs, which exhibit advanced magnetic properties when loaded with magnetic NPs, together with the advantages of biochar such as eco-friendliness and biodegradable raw materials including biomass waste, sugarcane bagasse, rice straw, peanut shells, and herb remains.^37,38^ For instance, Reddy *et al.* introduced two MBC systems with biodegradable raw materials, namely iron-loaded rice husk biochar (Fe-RHB) and coir pith biochar (Fe-CPB) for the decomposition of Acid Red 1 (AR1) organic dye, which resulted in nearly complete decolorization.^39^

Herein, we aim to focus on MCSs and their applications in the synthesis of different pharmaceuticals and degradation of organic pollutants (dyes and versatile drugs) with an overview of the latest related literature. Certainly, our purpose is to concisely review the current literature associated with catalytic systems consisting of magnetic components to pave the way for the synthesis of pharmaceutical compounds and their degradation. Also, we briefly summarize hybrid and multifunctional MCSs. Besides, we discuss the various properties of MCSs such as magnetization, reusability, structural stability, turnover number (TON), and turnover frequency (TOF).

## 2. Classification of the magnetic micro- and nanostructures

Here, a classification of MCSs is presented to give a qualified overview on catalytic systems implemented for the synthesis of pharmaceutical compounds to detail the unique advantages in each case.

### 2.1. Magnetic metal oxides

Extensive studies on metallic particles have demonstrated their excellent properties in the catalytic, optical, electronic, magnetic, and antibacterial fields.^40^ Moreover, metallic nanoparticles and metal oxides have attracted attention due to their unique structure, electrical conductivity and electrocatalytic activity.^41^ Gold NPs enhance the therapeutic effects against tumors with low toxicity.^42^ Besides, gold and silver particles are exploited in biomedicine and drug delivery.^43^ Silver NPs are applied in tissue engineering, coating medical implants, and boosting injury curing according to their antimicrobial and anti-inflammatory potential.^44^ The wide application diversity such as catalysis, preparation of nanocomposites, and production of electronic pieces of noble metallic NPs such as Ag, Au, Pd, Cu, and platinum group in pure or alloy form has persuaded scientists to focus on investigating these species.^40,45–48^

One of the most famous and widely used species with ferrimagnetic properties is iron oxide magnetic nanoparticles (Fe_3_O_4_ MNPs). Pure Fe_3_O_4_ NPs are not usually utilized in electrochemical techniques because pure metallic NPs do not have sufficient chemical stability in air and are immediately oxidized. Also, pure Fe_3_O_4_ MNPs tend to form large aggregates due to their intense dipole–dipole attraction. Thus, approaches for the protection of MNPs during their synthesis and applications are of great importance. These methods vary from grafting with organic parts to coating them with polymeric, organic, and inorganic layers such as silica or carbon. Furthermore, these coating layers promote the properties of MNP. Specifically, the chemical stability of NPs prevents their aggregation and agglomeration, their toxicity is reduced, and the effective interactions or coactions between NPs with other particles or various ligands are enhances. Magnetic nanoparticles can be placed next to other nanoparticles in electrocatalytic applications and sensors.^41^ MNPs are used in composite materials, which involves two main steps, *i.e.*, preparation of MNPs, and then surface modification. Fe_3_O_4_ MNPs have abundant surface hydroxyl groups, which are active to bind to other organic structures through covalent bonds.

**Fig. 1:**
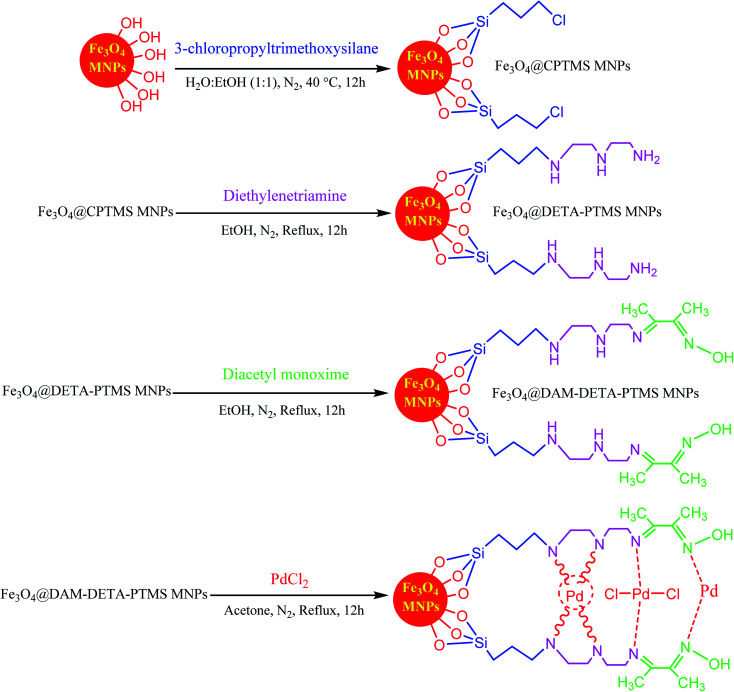
Synthesis and structure of Fe_3_O_4_@Pd–Schiff-base MNPs. This figure was adapted with permission from *Journal of Organometallic Chemistry*, 2019, **880**, 196–212.^49^

**Fig. 2:**
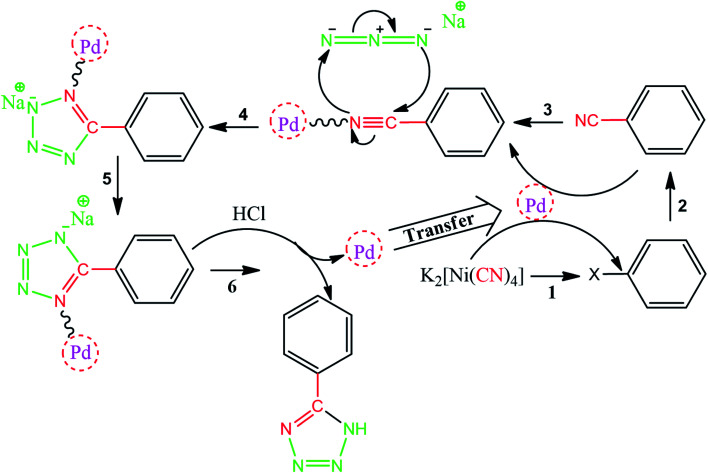
Suggested mechanism for the synthesis of 5-substituted 1*H* tetrazoles. This figure was adapted with permission from *Journal of Organometallic Chemistry*, 2019, **880**, 196–212.^49^

**Fig. 3:**
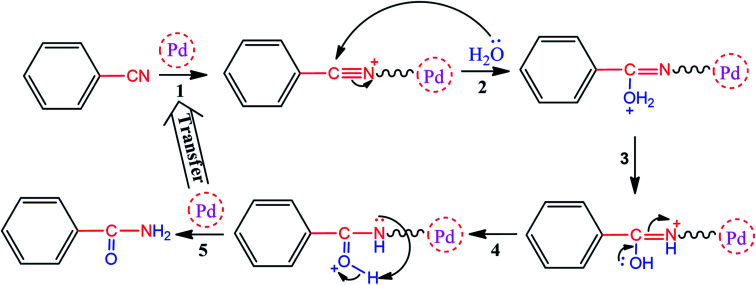
Proposed mechanism for the synthesis of benzamide. This figure was adapted by permission from *Journal of Organometallic Chemistry*, 2019, **880**, 196–212.^49^

As one of the brightest examples, Karimi Zarchi *et al.* reported a magnetic catalyst, palladium on surface-ameliorated Schiff base complex, Fe_3_O_4_@Pd–Schiff-base MNPs, as presented in Fig. 1.^49^ Due to the attached Pd complex, the magnetic saturation dropped from 62.10 emu g^−1^ for the Fe_3_O_4_ MNPs to 54.04 emu g^−1^ for the catalyst. This magnetic catalyst was utilized in the synthesis of 5-substituted 1*H* tetrazole (Fig. 2) and benzamide (Fig. 3). As a crucial fact, this reaction started from the insertion of the nontoxic K_2_[Ni(CN)_4_] inorganic azide source in aryl halide *via* a one-pot procedure. It should be highlighted that the metal leaching study for this system was negligible, as proven by hot filtration and ICP-AES tests, which confirmed the strong binding of palladium to the active sites on the surface of the catalyst. Consequently, due the low leaching and high stability and reusability of the catalyst, it could be recycled five times without any reduction in its catalytic performance. In some cases, 99% yield was obtained for 5-substituted 1*H* tetrazole and benzamide under the optimum condition of 5.7 h, which is comparable with long-time reactions in organic solvents.

**Fig. 4:**
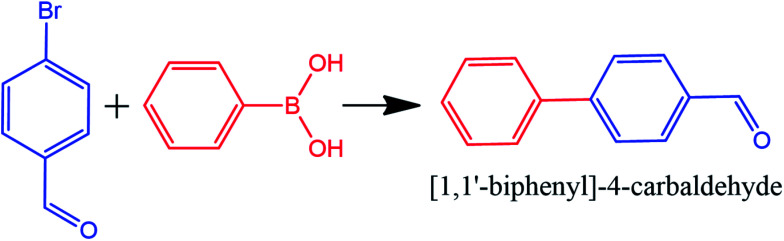
Suzuki cross-coupling reaction with Pd–Fe_3_O_4_/RGO heterogeneous catalyst. This figure was adapted with permission from H. A. Elazab, 2019 (DOI: 10.9767/bcrec.14.3.3518.478-489).^50^

The combination of pure metallic catalysts with magnetic agents through surface modification routes is considered in another approach. Herein, the active catalytic sites are together with the magnetic feature. As a related example, Elazab *et al.* proposed a highly active reduced graphene oxide-supported Pd/Fe_3_O_4_ NP catalyst with a uniform dispersion of palladium NPs on its surface. This catalyst exhibited very high catalytic activity under both batch and continuous reaction conditions with 100% conversion in the 4-bromobenzaldehyde Suzuki cross-coupling reaction with phenylboronic acid, as shown in Fig. 4.^50^

The structural defects in graphene sheets enhanced the catalytic activity and the interactions with anchored NPs. The remarkable benefits of adopting the flow chemistry method include a shorter reaction time and higher conversion rate and selectivity than the ordinary batch approach. As an important point, the reduction reaction conducted by microwave irradiation resulted in rapid heating of the reaction mixture. Palladium inhibited the agglomeration of the products during the graphene oxide reduction process.

**Fig. 5:**
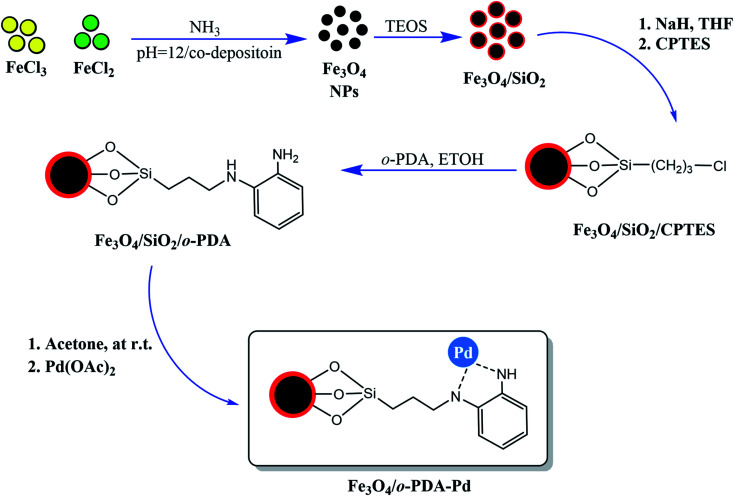
Preparation of Fe_3_O_4_/*o*-PDA–Pd heterogeneous magnetic nanocatalyst. This figure was adapted with permission from *Journal of Physics and Chemistry of Solids*, 2020, **136**, 109200.^17^

In another work, the focus was the core–shell structure of the Fe_3_O_4_ magnetic core and SiO_2_ protecting shell that, which was functionalized with *ortho*-phenylenediamine (PDA) and Pd ions to form (Fe_3_O_4_/*o*-PDA–Pd), as depicted in Fig. 5.^17^ A general fact that should be considered in the case of core–shell structures owning a magnetic core is that a stronger external magnetic field is required given that there is an inverse relationship between the core covering layers and its magnetic property.

MNPs are prepared *via* physical and chemical preparation routes. Physical methods such as ball milling can be divided into ordinary ball milling method^51^ and high-energy ball milling method.^52^ The chemical procedures for the preparation of MNPs include co-precipitation,^53^ hydrothermal,^54^ pyrolysis,^55^ sol–gel,^56^ microemulsion,^57^ sonochemical,^58^ electrodeposition,^59^ and polyol methods.^60^

**Fig. 6:**
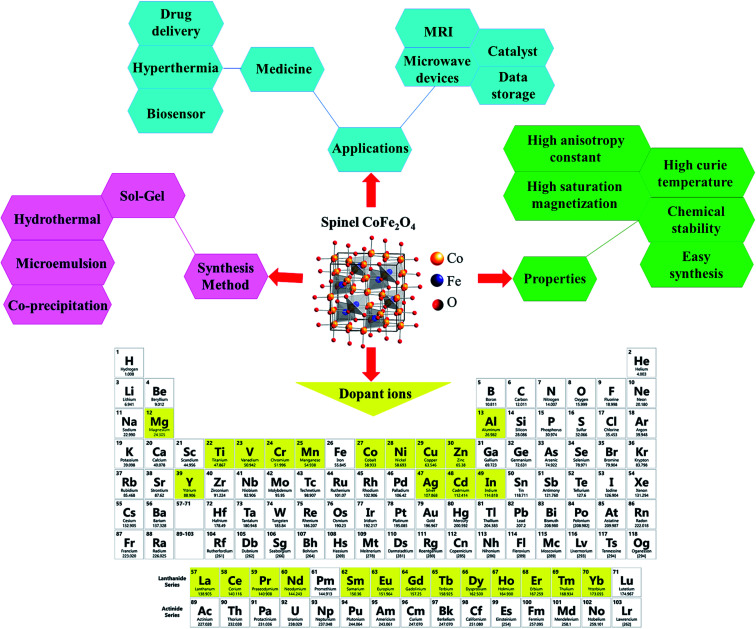
Characteristics, synthesis methods, and applications of CoFe_2_O_4_. This figure was adapted with permission from *Ceramics International*, 2020, **46**, 18391–18412.^68^

Ferrites as a member of ferrimagnetic ceramics with the general formula of MFe_2_O_4_ (M signifies bivalent metal ions including Mn, Fe, Co, Ni, Cu, and Zn) are well known because of their physical and magnetic properties, electrical resistance, high chemical stability, and low cost.^61–63^ According to the initial crystal lattice of ferrites, their structures are garnet, hexagonal, and spinel.^64–66^ Among these structures, normal and inverse spinel ferrites are very attractive. Cobalt and iron oxides have higher magnetic properties compared to other ferrites. The properties of cobalt ferrites can be altered by applying different methods such as chemical substitution, heat treatment, and sudden cooling.^67^Fig. 6 shows the characteristics, synthesis routes, and applications of CoFe_2_O_4_.^68^

**Fig. 7:**
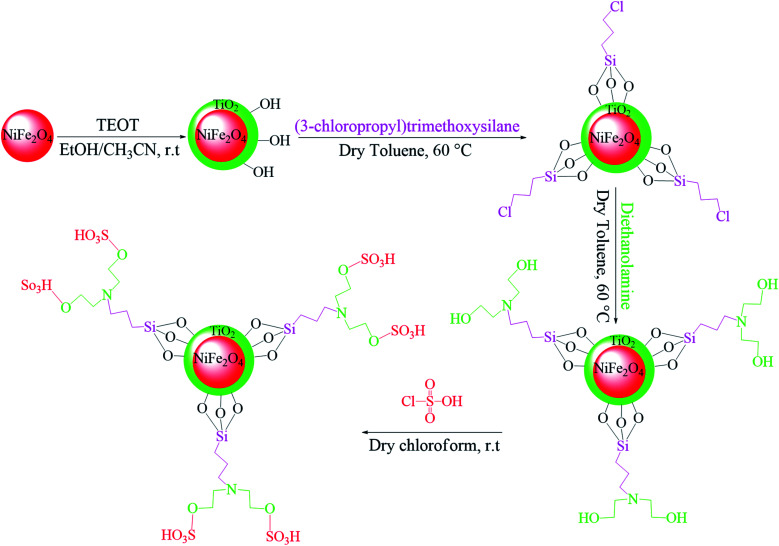
Approach for the preparation of the NiFe_2_O_4_@TiO_2_–SiO_2_–Pr–DEA–OSO_3_H nanocatalyst (r.t: room temperature). This figure was adapted with permission from *Chem. Sel*., 2019, **4**, 17–23.^71^

In the ferrites category, copper spinel ferrites (CuFe_2_O_4_) are appealing candidates due to their strong anisotropy, high coercivity, acceptable magnetic saturation, and excellent mechanical and chemical stabilities at high temperatures. The other relevant superiorities of CuFe_2_O_4_ MNPs include their simple modification, surface functionalization, and good reusability and catalytic activity.^69^ In the study by Maleki *et al.*, nanoscale ZnS/CuFe_2_O_4_ was used as a magnetic hybrid heterogeneous catalyst *via* the co-precipitation approach, which was retrieved by an external magnet from the reaction mixture. This magnetic hybrid composite catalyzed 2,4,5-triaryl-1*H*-imidazoles. The other advantages of this synthesized catalyst were its cost-effectiveness and reusability for five times in alternating cycles without any discernible reduction in its catalytic activity.^70^ As one of the precious instances of the catalytic function of NiFe_2_O_4_ for the synthesis of pharmaceutical components, Hamidinasab *et al.* prepared a magnetically recoverable organoacid-decorated NiFe_2_O_4_ nanocatalyst for the multicomponent synthesis of some phthalazine-trione and benzo[4,5] imidazo[1,2-*a*]pyrimidine derivatives, which complied with green chemistry protocols (Fig. 7).^71^ As previously stated, the abundant OH groups on TiO_2_-coated nickel ferrite NPs act as active sites to bond with (3-chloropropyl) trimethoxysilane. Subsequently, nucleophilic attack occurs between the chlorine leaving group on NiFe_2_O_4_@TiO_2_–chloropropylsilane and OH on diethanolamine (DEA) as a nucleophile. Lastly, another nucleophilic substitution takes place between the surface OH groups on nano-NiFe_2_O_4_@TiO_2_–SiO_2_–Pr–DEA and chlorine leaving group on chlorosulfonic acid to constitute the nano-NiFe_2_O_4_@TiO_2_–SiO_2_–Pr–DEA–OSO_3_H hybrid nanocatalyst. According to the formation of titania and other layers around the NiFe_2_O_4_ core, the magnetic saturation decreased from 35.1 to 11.1 emu g^−1^ at high magnetic fields up to 8000 Oe. Both nano-NiFe_2_O_4_@TiO_2_–SiO_2_–Pr–DEA–OSO_3_H and neat NiFe_2_O_4_ exhibited superparamagnetism. In this regard, it is reasonable to use neat NiFe_2_O_4_ as an inorganic magnetic catalyst with higher magnetic saturation and more convenient workup due to the work reported by Kaviyarasu *et al.*^72^ In their study, neat NiFe_2_O_4_ was prepared *via* the microwave combustion method (MCM) and conventional combustion method (CCM). The magnetic saturation value at 10 000 Oe magnetic field increased from 40.76 emu g^−1^ for CCM to 42.49 emu g^−1^ for MCM because of the narrow and limited distribution of NPs in MCM. Plenty of parameters affect saturation magnetization. Indeed, the magnetic saturation increases with the crystallinity of the NPs.^73^ On the contrary, as stated by Dai *et al.*, an increase in calcination temperature is inversely proportional to the saturation magnetization given that defects and strain are abundant in the product during the calcination process, although some are relieved throughout the procedure.^74^

**Fig. 8:**
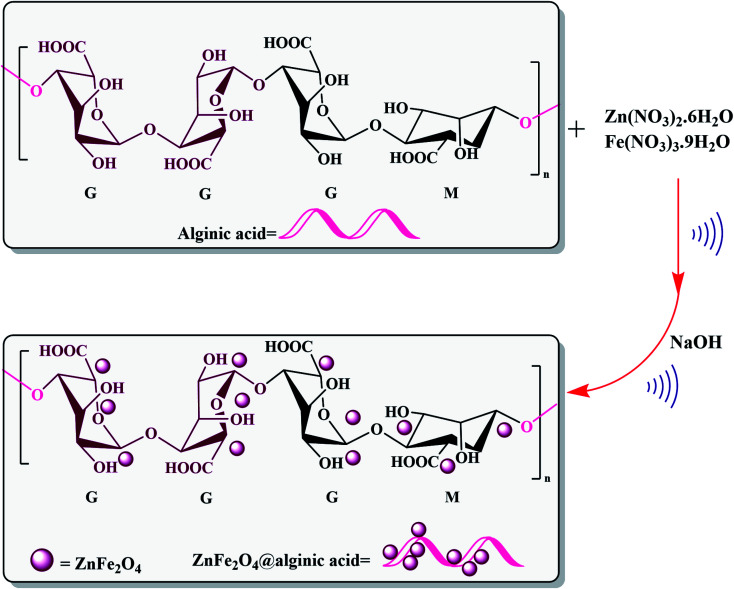
Synthesis of ZnFe_2_O_4_@alginic acid. This figure was adapted with permission from *Polyhedron*, 2019, **171**, 193–202.^75^

Elsewhere, as another type of MFe_2_O_4_, ZnFe_2_O_4_ MNPs and alginic acid (as a biopolymer) nanocomposite were recently synthesized, as shown in Fig. 8, and applied in an efficient catalyst for the practical synthesis of 2-amino-3-cyano-4*H*-pyran derivatives.^75^

The distinctive benefits of this process are mild conditions, short reaction time, facile workup, high yield, and green synthesis procedure. As a disadvantage, the magnetic saturation of the prepared catalyst (17.06 emu g^−1^) was lower than the neat ZnFe_2_O_4_ MNPs, which could be ascribed to the magnetically neutral alginic acid structure present in the catalyst. However, despite the decreased magnetic saturation of the catalyst, it could be simply separated from the reaction environment.

### 2.2. Magnetic frameworks

**Fig. 9:**
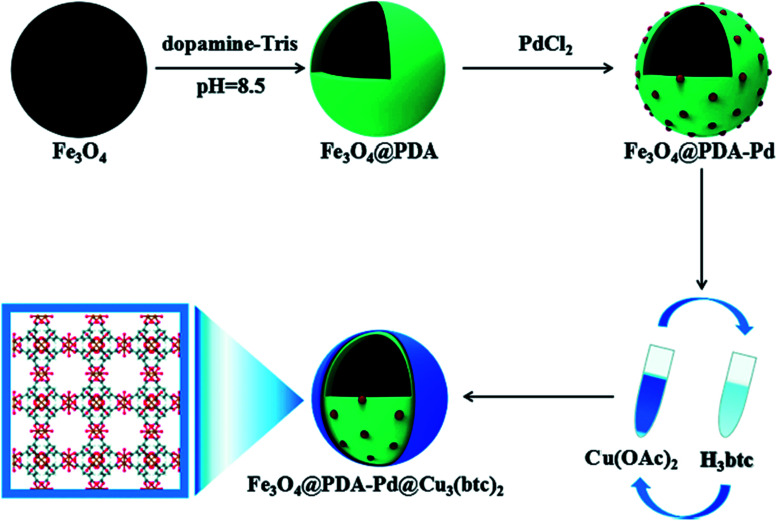
Schematic of the procedure for the synthesis of the Fe_3_O_4_@PDA–Pd@[Cu_3_(BTC)_2_] nanocomposite. This figure was adapted with permission from *ChemCatChem*, 2018, **10**, 1446–1454.^77^

Metal–organic frameworks (MOFs) are novel porous materials composed of ions or clusters of metals linked to organic ligands with coordination bonds. MOFs are not only magnificent adsorbents but also catalysts due to their consistent distribution of single-site or extremely small metal species, high porous structure, large surface area, and abundant active sites.^76^ Magnetic MOF nanocomposites are an example of materials with facile separation and improved catalytic activity. However, one of the main challenges that still need to be solved is how to assemble MOFs on the surface of MNPs without modifying their surface.^77^ The combination of Fe3O4 nanoparticles with Fe-MOFs endows the composite catalyst some advantages, such as easy magnetic separation, reusability, and other enhancements such as photo-Fenton performance. Fe_3_O_4_ nanoparticles can be *in situ* lodged into the MOF structure, acting as a magnetic core in magnetic MOF core–shell structures.^78^ Bian *et al.* synthesized a novel hierarchical core–shell Fe_3_O_4_@PDA–Pd@[Cu_3_(BTC)_2_] nanocomposite *via* a layer by layer assembly method, as shown in Fig. 9.^77^

There are also magnetic frameworks with inserted second-row transition metals. This inclusion extends the performance of these materials to various application ranges due to their advanced covalency, redox capability, and last-row spin–orbit coupling compared to their first-row analogs. Mononuclear niobium and molybdenum sites in MOFs display a wide variety of second-and third-row transition metals for stronger magnetic coupling in frameworks.^79^

The preferred method for the synthesis of magnetic MOFs is the solvothermal method, sometimes coupled with microwave and ultrasonication treatment.^78,80^ Recently, Espallargas *et al.* synthesized 2D Fe-based magnetic MOF nanosheets named MUV-1-X *via* a liquid exfoliation method. The resulting crystalline layers had favourable lateral size and thickness, which retained the structural magnetic properties.^81^

Besides, MOFs are well-defined solid structures to carry immobilized enzymes in a robust biocatalytic system. Enzymes are natural biocatalysts, demonstrating high selectivity and catalytic performances for the synthesis of chemicals and pharmaceuticals. Although the porosity of MOFs plays a vital role in their structure and performance, hierarchically porous MOFs introduce an adaptable pore distribution from the micro to meso-scale to provide better accessibility to immobilize large molecules such as enzymes. In the work by Zheng *et al.*, they have prepared a magnetic hierarchically porous core–shell Fe_3_O_4_@MOF structure through the formation of modulator-induced defects. Then, amidase was immobilized on this magnetic carrier. The properties of this magnetic MOF biocomposite were optimized, resulting in a high enzyme loading, high catalytic yield, thermal and storage stability, and reusability in comparison with the free enzyme and similar structures without hierarchical porosity.^82^ In another investigation, Zhang *et al.* focused their efforts on the preparation of a magnetic metal–organic framework, NiFe_2_O_4_@MOF-5.

**Fig. 10:**
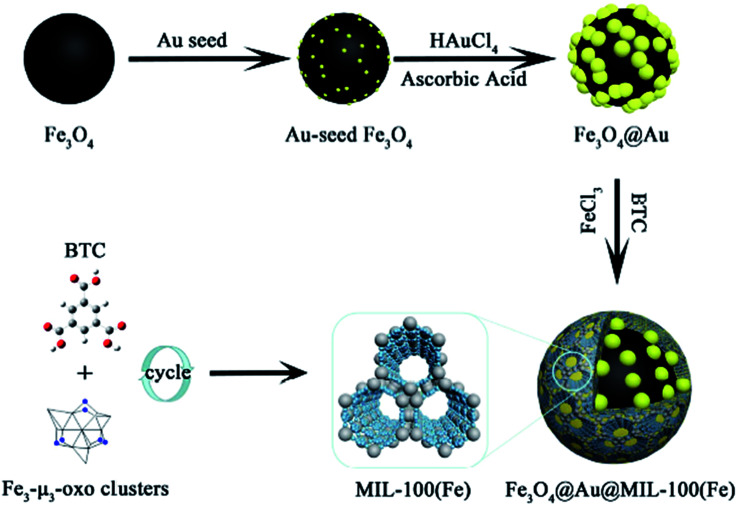
Illustration of the synthetic route to obtain magnetic MOF-based nanocatalysts. This figure was adapted with permission from *ACS Applied Materials & Interfaces*, 2018, **10**, 25726–25736.^85^

NiFe_2_O_4_ is a spinel ferrite structure with high saturation magnetization and strong chemical stability. The combination of ferrite and MOF resulted in excellent magnetic susceptibility, which allows easy separation and reusability.^83^ Song *et al.* reported the synthesis of the (Fe_3_O_4_@Au@MIL-100(Fe)) structure *via* a reaction including three successive steps. These steps included a solvothermal reaction, Au seed-induced growth and low-temperature cycling self-assembly, as shown in Fig. 10. This magnetic nanocatalyst acted as a peroxide mimic for the catalytic oxidation of the 3,3,5,5-tetramethylbenzidine (TMB) substrate. A prevalent non-devastating analytical technique used in chemical and biological analyses is surface-enhanced Raman scattering.^84^ The vibrational modes of a molecule show a unique and fingerprint-like spectrum in the SERS technique to show valuable inherent structural information. *In situ* SERS spectroscopy can be employed to monitor the whole reaction. However, MOFs themselves do not have excellent SERS enhancement ability. Thus, the use of MOF composites with noble metals can modify their signal enhancement ability. According to the distinctive structure and catalytic characteristics of an Fe-based magnetic MOF, under photo-irradiation with the assistance of ascorbic acid (AA), enhance photo-induced catalytic oxidation was achieved.^85^

Covalent organic frameworks (COFs) are organic porous structures, which have strong couplings among their building blocks. According to their characteristics including high chemical and thermal stability, structural resilience, and simple surface modification, COFs can be applied in various applications such as sensors, gas storage systems, catalytic approaches and many others. The density of COFs is lower than that of MOFs, resulting in enhanced stability in different acidic and basic pH and redox conditions. COFs have high sustainability to endure rough conditions without losing their crystallinity and orderly structure. COFs are one of the best candidates for heterogeneous catalysis. They can act as a host for MNPs in host–guest supramolecular structures. COFs are well isolated to prevent the agglomeration of MNPs and their large spatial pore distribution and channel permeability allow access to catalytically active substances as a nanoreactor. MNPs have desirable properties, and thus have received significant attention from scientists, ranging from effortless magnetic recovery, low toxicity, and the ability to have various morphologies to low cost. Thus, due to the excellent properties of MNPs and COFs, their combination as magnetic COFs uncovers a vast, fascinating world of materials.^86^

**Fig. 11:**
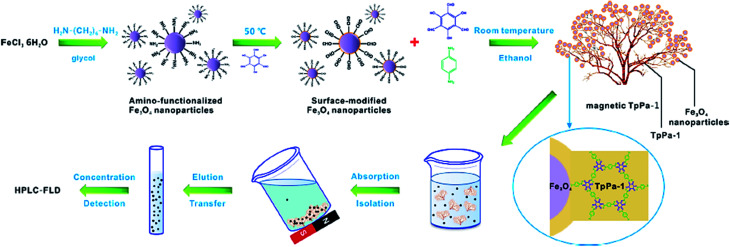
Schematic showing the synthesis and function of the bouquet-like magnetic TpPa-1 sorbent. This figure adapted with permission from *ACS Applied Materials & Interfaces*, 2017, **9**, 2959–2965.^87^

Additionally, MNPs can enhance the efficiency of the procedure and reusability of the catalyst. Cai *et al.* synthesized a unique *Gypsophila* bouquet-shaped magnetic COF through facile mechanochemical grinding followed by crystallization. In this work, amino-functionalized Fe_3_O_4_ nanoparticles were grafted to the Tp monomer (COF monomer). Then, Tp and Pa-1 (another monomer) were combined, resulting the formation of the magnetic TpPa-1. This magnetic COF exhibited superparamagnetic property and a large surface area due to its porous structure. It was used to extract trace analytes such as polycyclic aromatic hydrocarbons (PAHs) from ecological samples. The preparation and function of the bouquet-like magnetic TpPa-1 sorbent are illustrated in Fig. 11.^87^

### 2.3. Natural-based magnetic systems

Considering that green synthesis and biocompatibility are vital aspects in catalytic systems, the presence of natural-based environmentally friendly magnetic compounds appear to be suitable for scaling up procedures, simplicity in final purification processes and use in industrial applications. Versatile natural species such as clays and polymers have become the focus of scientists after biocompatibility.^30^ Intrinsically magnetic natural species such as pumice, magnetite, hematite, ilmenite, and pyrrhotite (monoclinic Fe_7_S_8_) are utilized in competent catalytic systems. Magnetite, iron(ii and iii) oxide, a with brownish-black color, has the highest magnetic properties among the natural-based minerals. The chemical composition of pure hematite (Fe_2_O_3_) consists of about 70% iron and 30% oxygen by weight. Magnetite is used in magnetic-derived catalysts in various processes. Magnetite NPs are employed in biomedical, environmental, and ferrofluid applications.^88–90^ However, unlike magnetite, hematite has weak attraction to a magnetic field. Hematite is applied as a pigment mineral, healing stone, and catalyst with magnetic separation ability.^91^ Additionally, the paramagnetic ilmenite (FeTiO_3_) and the ferrimagnetic pyrrhotite (monoclinic Fe_7_S_8_) have emerged as great magnetically separable catalysts in different reactions.^92,93^ Volcanic pumice magnetic particles (VPMPs) are light-colored, highly porous volcanic rocks with an intrinsic magnetic property of 30 emu g^−1^. VPMPs are biocompatible and have high surface functionalization capacity.^94^ Furthermore, the physical properties of VPMPs, such as lightweight porous structures, are well suited for filtration and concrete construction applications. The chemical structure of pumice consists of a silica network, aluminum oxide, and aluminum silicate, and similar to other minerals, it has small amounts of the other metal oxides. Also, pumice can be functionalized with other species due to its chemical structure and abundant OH groups.^95^

### 2.4. Hybrid micro and nanoscale magnetic composites

Recently, organic–inorganic hybrid materials have been implicated as heterogeneous catalysts given that they exhibit the advantages of both homogeneous and heterogeneous catalysts in organic synthesis.^96^ The chemically bonded hybrid materials have been described as materials with covalent or partially covalent bonds. Hybrid catalysts consist of two distinguished phases with completely different properties and functionalities, which have synergistic combinations to develop overall catalytic properties.^97^

**Fig. 12:**
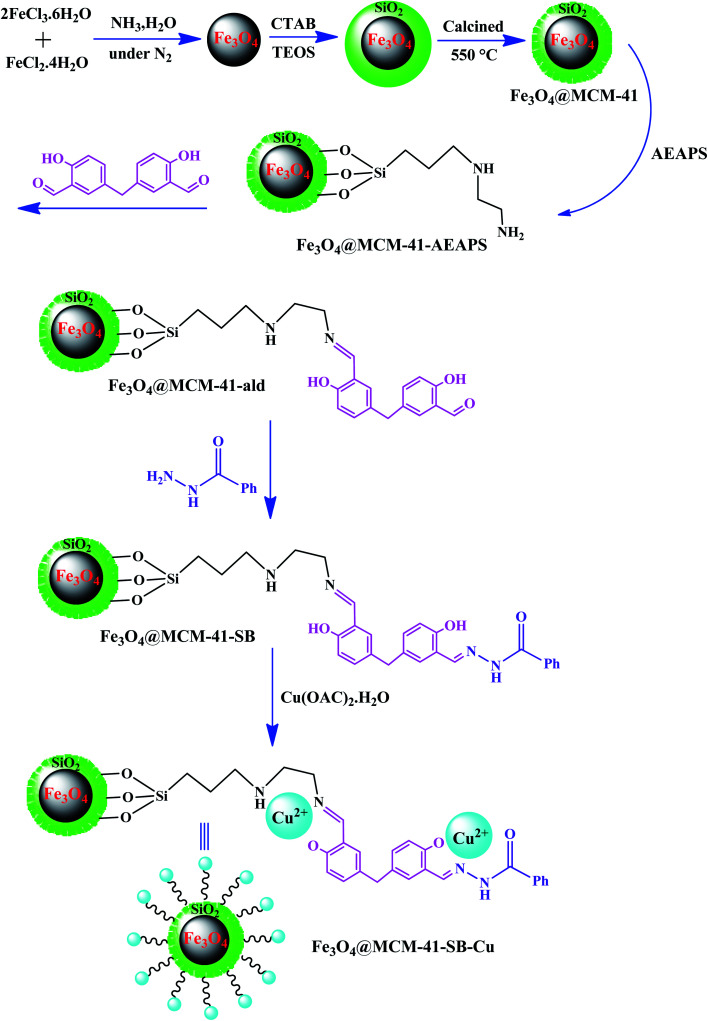
Synthetic route of functionalized magnetic Fe_3_O_4_@MCM-41–SB–Cu hybrid catalyst. This figure was adapted with permission from *Applied Organometallic Chemistry*, 2020, **34**, e5572.^98^

In one of the most recent reports, Sedaghat *et al.* introduced an efficient hybrid catalyst with antibacterial property for the synthesis of 5-substituted-1*H*-tetrazoles.^98^ In this regard, a Cu^2+^–Schiff base complex was anchored on magnetic mesoporous silica NPs to form Fe_3_O_4_@MCM-41–SB–Cu (Fig. 12). The possibility to load high amounts of copper on the catalyst is due to the presence of many coordination sites in the bis-Schiff base ligand. Besides, a coordination bond is formed between the oxygen and nitrogen atoms of the supported Schiff base and Cu^2+^ ions. Significantly, the long catalyst linkers in this study resulted in easy access by the reactants to the active sites of the catalyst compared to the studies with short linkers.

From a mechanistic point of view, during the synthesis of 5-substituted-1*H*-tetrazoles, the interaction between Cu^2+^ ions and oxygen atoms of the aldehyde and nitrogen atoms of the nitrile increases the electrophilicity of the aldehyde and nitrile, respectively, which promotes further interactions. The high catalytic performance and non-toxicity of the Cu catalysts led to excellent catalytic yields (69–95%) in this reaction.

**Fig. 13:**
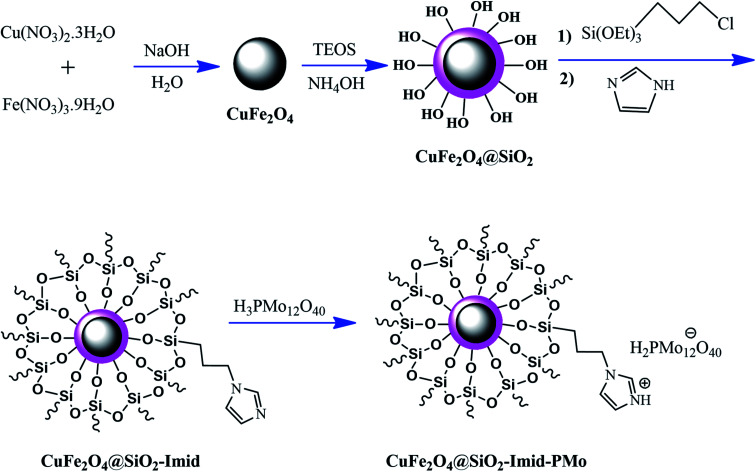
Synthesis of CuFe_2_O_4_@Si-Imid-PMo catalyst. This figure was adapted with permission from: *Quarterly Journal of Iranian Chemical Communication*, 2019, **7**, 271–282.^99^

Ferrites can also act as the magnetic core of hybrid catalysts. As an example, a hybrid nanocomposite of organoacid-decorated NiFe_2_O_4_ was prepared. The advantages of the used MNPs included not only their superparamagnetic behavior and effortless separation from the reaction environment, but also considerable surface area, low toxicity, good stability, and easy surface functionalization.^71^ Another example of ferrites is CuFe_2_O_4_@Si–Imid–PMo, which consists of an acidic ionic liquid based on the imidazolium cation and phosphomolybdic acid anion, both immobilized on the CuFe_2_O_4_@SiO_2_ core–shell magnetic structure (Fig. 13).^99^ This catalyst was applied in the synthesis of 2,4,5-trisubstituted imidazole derivatives. Matching with previous studies involving several functionalization steps on a magnetic core, the magnetic saturation of this catalyst decreased from 25.85 to 24.1 emu g^−1^ under an applied magnetic field of 10^4^ Oe compared to the neat CuFe_2_O_4_, which is ascribed to its non-magnetic silica shell and immobilized ionic liquid. However, the magnetization of this catalyst was sufficient for easy magnetic separation. The CuFe_2_O_4_@Si–Imid–PMo catalyst improved the production of the intermediate during the reaction, while it increased the electrophilicity of the electrophiles according to its role as a Brønsted acid center.

**Fig. 14:**
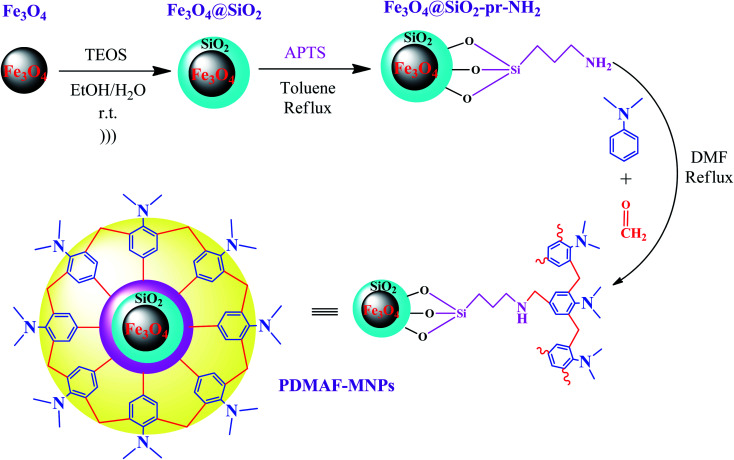
Synthesis of poly *N*,*N*-dimethylaniline-formaldehyde supported on silica-coated Fe_3_O_4_ magnetic nanoparticles (PDMAF-MNPs). This figure was adapted with permission from *Research on Chemical Intermediates*, 2020, **46**, 1629–1643.^100^

In a recent study reported by Bodaghifard *et al.*, a magnetic core–shell structure was functionalized by polymer for the green synthesis of 2-amino-3-cyanopyridine derivatives.^100^ This hybrid heterogeneous catalyst contained poly *N*,*N*-dimethylaniline-formaldehyde supported on Fe_3_O_4_@SiO_2_ (PDMAF-MNPs), as depicted in Fig. 14. It should be noted that the reduction in magnetic saturation compared to its pure magnetic core is much greater in the case of polymer linkers compared to other short organic likers, as stated in a previous study. According to the silica shell and polymeric linker around Fe_3_O_4_, the magnetic saturation declined from 53.5 to 31.1 emu g^−1^ in high magnetic fields up to 8000.0 Oe. The unpaired electron pairs of nitrogen in this eco-friendly catalyst expedited the Knoevenagel condensation of benzaldehyde and malononitrile and also Michael addition of cyclohexanone by taking the hydrogen of its components to form intermediates and adducts in the synthesis of 2-amino-3-cyanopyridine derivatives. This catalyst resulted in efficient isolated yields in the range of 74–93%. This retrievable catalyst was recycled six times with no distinctive structural alteration and change in its catalytic behavior.

**Fig. 15:**
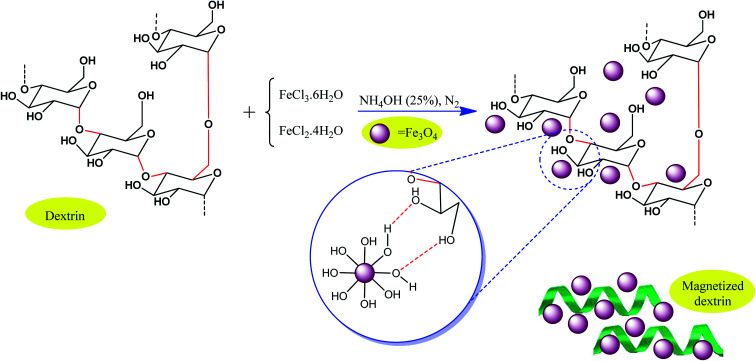
Process for the preparation of magnetic dextrin. This figure was adapted with permission from *Materials Science and Engineering: C*, 2020, **109**, 110502.^101^

As another strategy, conventional biocompatible and naturally occurring catalysts are combined with magnetic particles through surface modification approaches. Consequently, the active sites of the catalyst also exhibit magnetic property. For example, Maleki *et al.* designed an efficient magnetic hybrid catalyst, as shown in Fig. 15, for the synthesis of biologically active polyhydroquinoline derivatives.^101^ In the design of natural polymer-based hybrid catalysts, dextrin, which originates from natural polysaccharide resources, is beneficial due to its many effective advantages such as abundant active functional groups, non-toxicity and biocompatibility, availability, environmentally friendly nature, ability to prevent unwanted side reactions, and stereoselectivity in some organic reactions. Thus, the question arises, how can magnetic dextrin effectively act as a hybrid biocatalyst in pharmaceutical synthesis reactions?

On the one hand, the convenient separation of the catalyst is ascribed to iron oxide MNPs with acceptable particle distribution, high surface area to volume ratio, and superparamagnetic feature. On the other hand, its abundant reactive functional groups give the catalyst the ability to interact with other organic components *via* hydrogen bonds derived from the its hydroxyl groups and the electronegative atoms of the functional groups in the other material. Accordingly, the electrophilicity of the designed materials would be enhanced, finally facilitating the reaction. The synthesis of polyhydroquinoline derivatives *via* the asymmetric Hantzsch reaction took advantage of this catalyst to overcome the limitations in previous studies such as tedious workup process, harsh reaction conditions, and unsafe catalysts by presenting an appropriate yield (70–95%), short reaction time (15–45 min), and five times recycling. Also, polyhydroquinoline derivatives have numerous pharmaceutical applications, for example, vasodilators, anti-atherosclerotic, hepatoprotective, antitumor, bronchodilator, antidiabetic, and calcium channel blockers.^102^

**Fig. 16:**
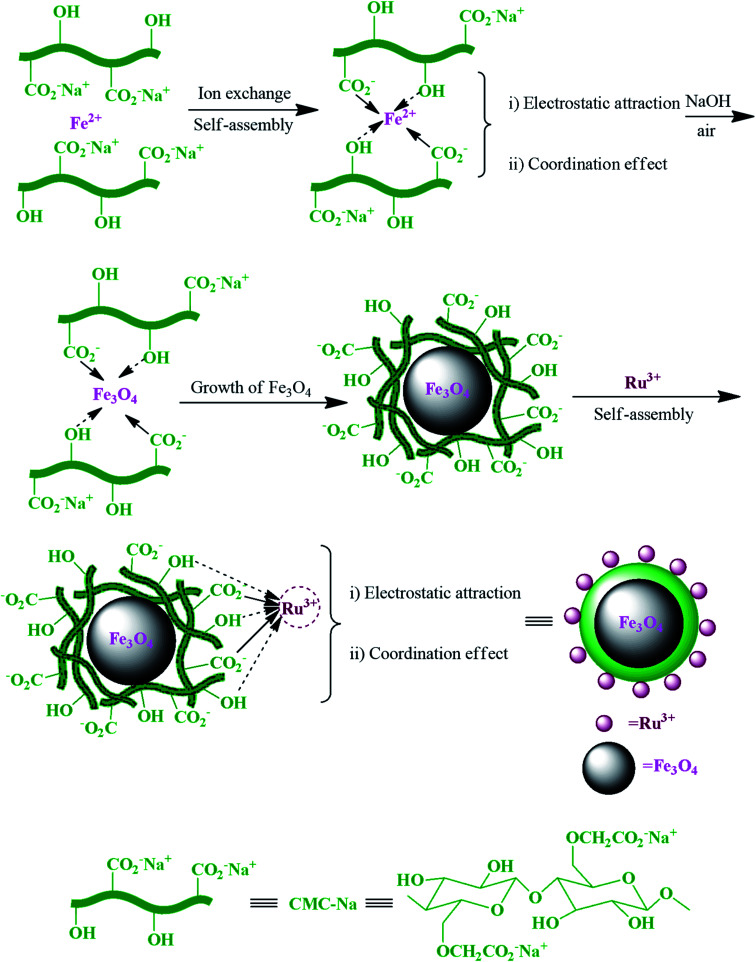
Schematic of the preparation of magnetic Ru^III^@CMC/Fe_3_O_4_ organic/inorganic hybrid catalyst *via* self-assembly. This figure was adapted with permission from: *Molecular Diversity*, 2019, **23**, 421–442.^103^

In another study, Ru^3+^ and carboxymethylcellulose (CMC) in the magnetic Ru^III^@CMC/Fe_3_O_4_ hybrid catalyst (Fig. 16) exhibited a noticeable synergistic effect during each step in the synthesis of polyhydroquinoline derivatives.^103^

**Fig. 17:**
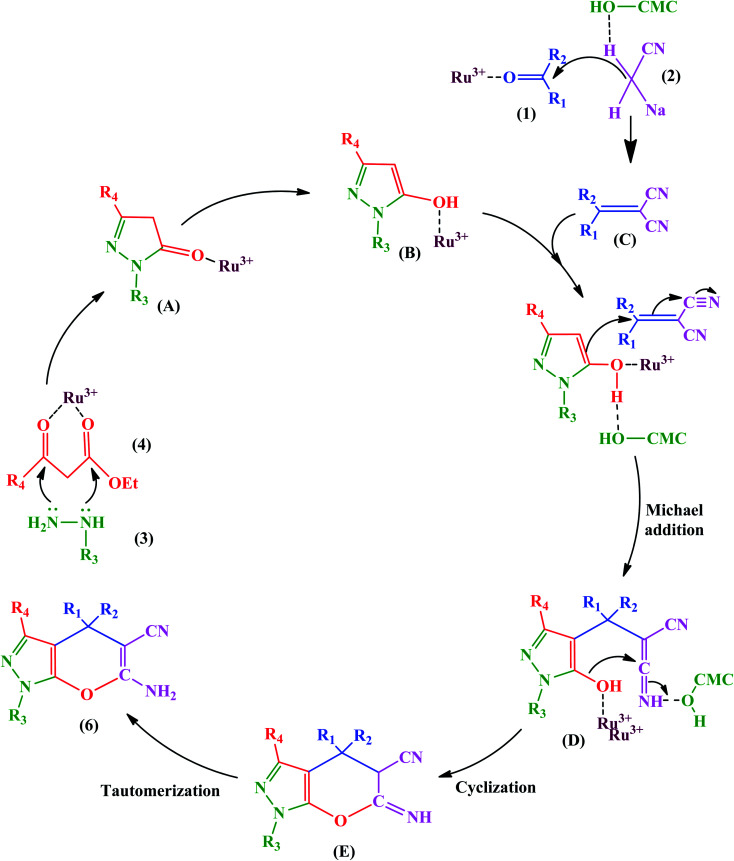
Proposed mechanism for synthesis of pyrano[2,3-*c*]pyrazoles. This figure was adapted with permission from *Molecular Diversity*, 2019, **23**, 421–442.^103^

This advantage of the above-mentioned hybrid catalyst is attributed to the Ru^3+^ Lewis acid, which can be chelated with the carboxyl and free hydroxyl groups of CMCs. Ru^3+^ activated β-keto ester (4) to facilitate further nucleophilic attack in the condensation of hydrazine (3). Also, it acts as a center through which an electron transfers to give the enol (B) form from keto (A). Besides, Ru^3+^ and hydrogen bonding advance the Knoevenagel condensation of a carbonyl compound (1) with malononitrile (2). It should be noted that the Ru^3+^ and hydroxyl groups of CMCs are active catalytic sites to promote the Michael addition of the reaction intermediates (B and C), as displayed in Fig. 17.

**Fig. 18:**
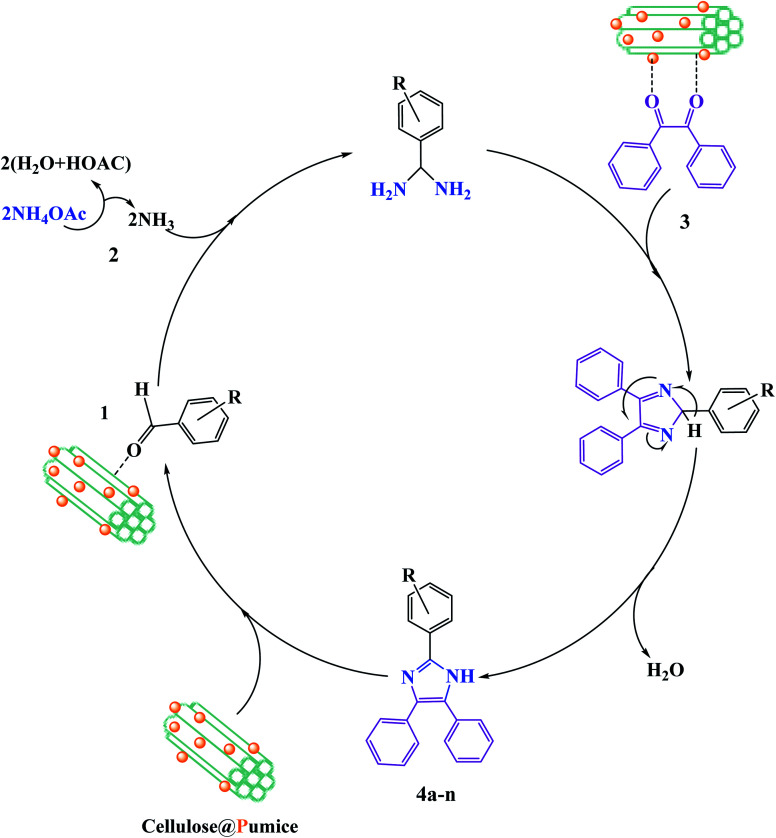
Suggested reaction mechanism for the synthesis of 2,4,5-triarylimidazoles (4a–n) utilizing a magnetic cellulose/pumice hybrid catalyst. This figure was adapted with permission from *Journal of Physics and Chemistry of Solids*, 2020, **142**, 109443.^94^

In the recent report on the use of hybrid catalysts for the synthesis of pharmaceuticals by Maleki *et al.*, they demonstrated the use of an eco-friendly solid-state hybrid catalyst.^94^ Specifically, in this study, they successfully demonstrated the synergistic impacts of ultrasonic waves and a natural-based magnetic cellulose/pumice hybrid catalyst for the synthesis of 2,4,5-triarylimidazoles. The mechanism for the synthesis of imidazole with respect to the role of the catalyst is shown in Fig. 18. The hydroxyl groups on cellulose activate the carbonyl group of benzaldehyde (1) and benzil (3) to further form intermediate I and II, respectively. According to the physical aspects, the highly porous structure of pumice created good electronic interactions between the components due to its very high surface area. Finally, because of the magnetic property of pumice, the catalyst was recycled for ten successive runs without any significant loss in its catalytic functionality.

Reportedly, a heterogeneous hybrid nanocomposite was synthesized *via* the co-precipitation method. Although the catalytic activity of each component of the composite was not significant, the hybrid structure showed much higher activities due to the composition of the materials. Alternatively, the reported hybrid structure offers characteristic properties and applications that are not achievable in each component alone. The synergistic effect of the metals and metal oxides in a hybrid catalyst scaffold expedites the organic reaction more efficiently.^70,104^Table 1 summarizes the information of some efficient magnetic catalytic systems, highlighting their target organic reactions and reaction yields.

**Table 1:** Brief information of some hybrid micro-and nanoscale magnetic composites

Entry	Catalyst	Function	Yield^a^ (%)	Ref.
1	TPP@CuhNfs^b^ and TPP@CohNfs^c^	Hydrogenation of nitrobenzenes	52–98%	105
			38–98%	
2	Fe_3_O_4_/PVA-10%Ag	Reduction of nitrobenzene derivatives	89–99	106
3	MNPs@C/UV/PMS^d^	Catalytic oxidative degradation of acetaminophen	97.4%	107
4	CoFe@rGO	Epoxide ring opening reaction with various aromatic amines of cyclohexene oxide	86–94%	108
		Cyclopentane oxide	85–94%	
		Styrene oxide	82–92%	
5	Fe_3_O_4_@Alg^e^@CPTMS^f^@Arg^g^	Synthesis of pyrazole derivatives	90–97%	109
6	[FSRN][H_2_PO_4_]^h^	Synthesis of pyrimido[4,5-*b*]quinolines	79–96%	110
7	γFe_2_O_3_@Sh^i^@Cu_2_O	Synthesis of 1,4-disubstituted-1,2,3-triazoles	50–98%	111
8	Fe_3_O_4_@Cu–β-CD^j^	Synthesis of dihydropyrano[2,3-*c*]pyrazoles	89–98%	112
9	Pd@GO^k^/Fe_3_O_4_/PAA^l^/DCA^m^	Sonogashira reaction of various halides with terminal alkynes	80–97%	113
10	ZnS–ZnFe_2_O_4_	Synthesis of 2,4,5-triaryl-1*H*-imidazoles	51–95%	114
11	Pd@CS^n^–CD^o^–MGQDs^p^	Hydrogenation of nitro compounds	80–97%	115
12	α-Fe_2_O_3_@Hap^q^@Cu	Synthesis of 1,4-disubstitued triazoles	40–97%	116
13	Fe_3_O_4_@SiO_2_–guanidine–poly acrylic acid	Synthesis of 4*H*-benzo[*b*]pyrans and dihydropyrano[*c*]chromenes	95–98%	117
14	Ferroferric oxide nanocatalyst@mesoporous activated carbon	Degradation of acetaminophen	98.6%	118
15	MCM41–Pr–THEIC^r^	Synthesis of 9-(aryl)-3,3,6,6-tetramethyl-3,4,6,7,9,10-hexahydroacridine-1,8(2*H*,5*H*)-dione derivatives	68–92%	119
16	Fe–DPMP^s^	Synthesis of tri-substituted imidazole derivatives	65–92%	120
17	Fe_3_O_4_@xanthan gum	2-Amino-3-cyano-4*H*-pyran derivatives	84–96%	121
18	Pd NPs@Fe_3_O_4_/chitosan/pumice hybrid beads	Cyanation of aryl halides	80–98%	122
19	ZnO/g-C_3_N_4_	Synthesis of biologically interesting small molecules of thiazolidinones	57–97%	123
20	Pd@Bipy–PMO^t^	Suzuki cross-coupling reactions of various aryl halides and bromic acid substrates	6–98%	124

a Isolated yield.

b Tetraphenyl porphyrin copper hybrid nanoflowers.

c Tetraphenyl porphyrin cobalt hybrid nanoflowers.

d Peroxymonosulfate.

e Alginate.

f 3-Chloropropyltrimethoxysilane.

g l-Arginine.

h [Fe_3_O_4_@–SiO_2_@R-NHMe_2_][H_2_PO_4_].

i Shilajit.

j β-Cyclodextrin.

k Graphene oxide.

l Polyacrylamide.

m Dicyandiamide.

n Chitosan.

o Cyclodextrin.

p Magnetic graphene quantum dots.

q Hydroxyapatite.

r 1,3,5-Tris(2-hydroxyethyl) isocyanurate-1,3-propylene covalently functionalized MCM-41.

s Diethylenetriamine penta.

t Palladium-containing dicationic bipyridinium-supported periodic mesoporous organosilica (PMO).

## 3. Application of micro and nanoscale magnetic catalytic systems in the degradation of pharmaceutical compounds

**Fig. 19:**
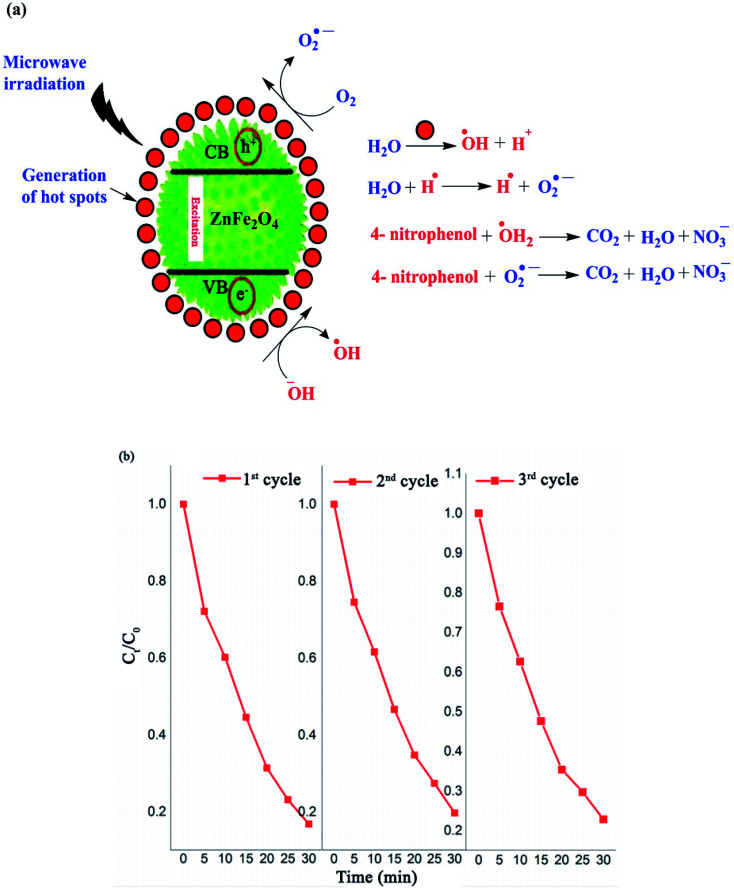
(a) Mechanism for the degradation of 4-nitrophenol using ZnMn_2_O_4_ catalyst. (b) Recyclability of spinel ZnMn_2_O_4_ absorbent during three degradation cycles under microwave irradiation. This figure was adapted with permission from *Journal of Materials Research and Technology*, 2020, **9**, 9709–9719.^135^

In recent decades, the discharge of toxic substances, including inorganic heavy metals and organic contaminants such as pesticides, herbicides, dyes and pharmaceuticals in water and sewage has become major human and ecology issues.^125–128^ Thus, a wide variety of water decontamination methods utilizing micro-and nano-adsorbents and/or photocatalysts have been developed.^129–131^ In addition, microwave- and ultrasonic-assisted pollutants catalysis approaches have gained wide interest in this field due to their synergistic effect of converging the dispersed microwave and ultrasound fields onto the reactive sites of the catalyst.^60,132,133^ These synergetic methods produce thermal or discharge effects around the catalyst, which lead to the *in situ* degradation of contaminants.^134^ Generally, microwave technology reduces the activation energy and organic reaction time due to the following reasons: firstly, the catalysts absorb microwaves to produce a thermal effect, which can create abundant hot spots to speed up organic reactions. Secondly, the coupling effects of microwaves and catalysts that have high wave absorption capacity result in a non-thermal effect, which can generate hydroxyl radicals with strong oxidation potential, leading to an enhancement in the oxidation rate of organic materials. As the most recent example, Riaz *et al.* prepared MnO_2_ nanorods and ZnMn_2_O_4_ nanostructures with hexagonal shapes for the microwave-assisted catalytic decomposition of 4-nitrophenol (*p*-NP) solution.^135^ ZnMn_2_O_4_ exhibited a high degradation efficiency of up to 85% in just 30 min. As displayed in Fig. 19a, similar to the photocatalytic degradation conducted under visible light, hot spots with high temperatures are produced on the ZnMn_2_O_4_ absorbent upon exposure to microwave irradiation. The water and dissolved O_2_ molecules produce ˙OH and H˙, and O_2_˙^−^ radicals by the heat absorbance from the hot spots, respectively.

Similarly, the ZnMn_2_O_4_ absorbent contributes to producing more ˙OH and O_2_˙^−^ radicals. The electrons in the valence band (VB) of the ZnMn_2_O_4_ absorbent are excited and transported to the conduction band (CB), which causes holes (h^+^) in the VB and electrons (e^−^) in the CB. The holes oxidize H_2_O to produce ˙OH and the electrons react with dissolved O_2_ to produce O_2_˙^−^. Eventually, this mechanism process facilitates the degradation of *p*-NP to CO_2_, H_2_O, and nitrate. According to the scavenging experiment results, it was found that the produced ˙OH radicals acted as the major active species in the decomposition compared to O_2_˙^−^. Besides, the ZnMn_2_O_4_ absorbent had a slight reduction in activity (from 83% to 79%) after three successive *p*-NP microwave-assisted degradation cycles, as shown in Fig. 19b.

**Fig. 20:**
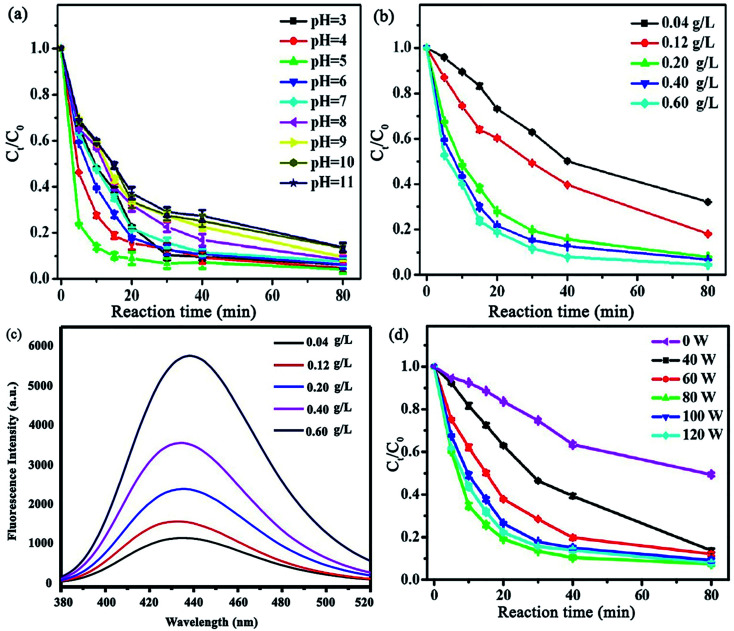
Effect of various parameters on TC removal: (a) solution pH, (b) Fe/N–C-2 concentration, and (c) influence of Fe/N–C-2 concentration (g L^−1^) on ˙OH production utilizing a terephthalic acid fluorescence probe. (Reaction conditions: 60 mM terephthalic acid concentration; 60 mM H_2_O_2_; reaction temperature: 25 °C; 80 W US power; and 80 min reaction time). (d) Ultrasonic power. This figure was adapted with permission from: *ACS Omega*, 2018, **3**, 15870–15878.^136^

Another study was devoted to investigating the synergistic effect of ultrasound irradiation power, catalyst dosage, solution pH, *etc.* on tetracycline (TC) ultrasound-assisted degradation using Fe/N–C-*x* hybrid/H_2_O_2_ Fenton-like catalysts (*x* stands for iron salt molar ratio (Fe(NO_3_)_3_·9H_2_O)).^136^ Unlike previous reports, the removal of TC decreased from 93.8 to only 86.3 in 80 min by altering the pH from 3 to 11, indicating that this catalyst has high removal efficiencies in a wide pH range (Fig. 20a). The TC degradation increased concurrently with an increase in Fe/N–C-2 concentration, which was attributed to the generation of more ˙OH radicals, resulting from the decomposition of H_2_O_2_ (Fig. 20b). Terephthalic acid, as a fluorescence probe, was employed to authenticate the amount of ˙OH radicals produced. A slight increase in the fluorescence response was assigned to the hydroxyl radicals produced by Fe/N–C-2 catalysis (Fig. 20c). Given that one of the most effective parameters to achieve superior degradation efficiencies is ultrasound irradiation, different ultrasonic powers (0, 40, 60, 80, 100, and 120 W) were applied to monitor their effects on the system (Fig. 20d). The increase in ultrasonic power was observed to be directly proportional to the TC degradation efficiency, confirming the synergistic catalytic effect of the Fe/N–C-2 catalyst and ultrasound waves.

Moreover, consecutive ultrasound exposure removed the byproducts; thus, the surface of the catalyst was free for further reactions. Furthermore, a higher ultrasonic power generates more cavitational bubbles with extremely high pressure and temperature, which collapse and produce solution turbulence, leading to elevated intensity of mixing between the catalyst and contaminant.^137^ Nevertheless, it should be noted that an additional increase in ultrasonic power resulted in lower TC degradation because the maximum growth and collapse of the cavitation bubbles occur together with the compression cycle in the cavitation process, resulting in attenuated cavitation action at high ultrasound irradiation intensity. Accordingly, 80 W was chosen as the optimized ultrasonic power.

Precisely, various magnetic catalytic systems and the pioneering and most powerful photocatalysts are discussed briefly. As the first and foremost example of photocatalysts, TiO_2_ is well-known due to its chemical stability, non-toxicity, and comparatively low cost. As stated above, photocatalytic degradation is triggered when a photon is sufficiently excited to over the bandgap energy value of a semiconductor. Semiconductor materials with a wide bandgap, such as TiO_2_ with a bandgap of 3.2 eV in the anatase state, are less active under visible light irradiation.^138^ UV irradiation is the most potent energy source for the degradation of pollutants; however, it poses a risk to human health and has detrimental effects on the eyes.^139^ Thus, various strategies have been developed to produce visible light-active TiO_2_ photocatalysts such as metal doping,^140^ non-metal doping,^141^ codoping with different semiconductors,^142^ and dye sensitization.^143^ The presence of metal dopants in the structure of TiO_2_ delays the recombination of charge carriers by electron trapping, changing its bandgap energy and physical characteristics.

**Fig. 21:**
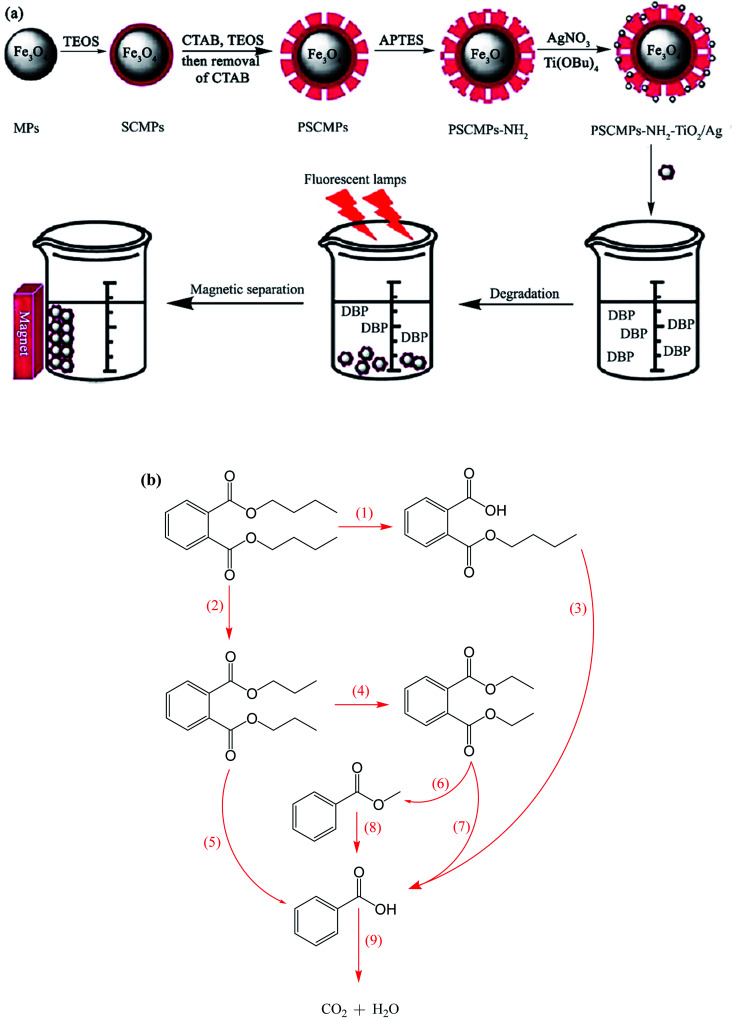
(a) Synthesis of magnetic photocatalysts and their application for the degradation of dibutyl phthalate under visible irradiation. (b) Suggested photocatalytic degradation routes for DBP. This figure was adapted with permission from *Water Science and Technology*, 2020, **81**, 790–800.^144^

For instance, Ag co-doped TiO_2_ nanostructures grafted on Fe_3_O_4_ NPs were synthesized through a facile and affordable co-precipitation approach. They were applied for the degradation of dibutyl phthalate (DBP), which is a toxic ecological pollutant (Fig. 21a).^144^ The degradation intermediates such as butyl phthalate, diethyl phthalate, dipropyl phthalate, methyl benzoate, and benzoic acid were detected by gas chromatography/mass spectrometry (GC-MS) analysis, and the two possible decomposition routes are illustrated in Fig. 21b. During the reaction, the generated excitons by light stimulation of TiO_2_ are transferred to the surface of the catalyst to react with OH^−^, O_2_, H_2_O, and other species. The ˙OH radicals break DBP in two possible ways, as follows: the first way is to break the C–O bond of DBP to form monobutyl phthalate accompanied by the breakage of another C–O bond to produce phthalic acid.

The further decomposition of phthalic acid generates benzoic acid, which breaks into CO_2_ and H_2_O. The second path is the degradation of the DBP molecules from various sides of its carbon chain to form dipropyl phthalate and diethyl phthalate as the main products. In the subsequent reaction stage, methyl benzoate and benzoic acid are generated by the actions of ˙OH radical. Similarly, benzoic acid degrades to CO_2_ and H_2_O. The DBP degradation efficiency reached 74%, which remained almost the same after five cycles, proving the high stability and reusability of the catalyst.

**Fig. 22:**
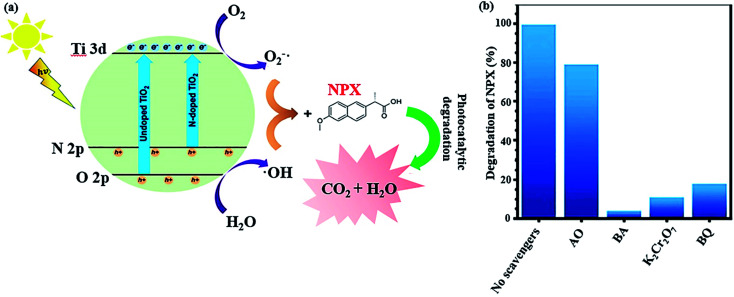
(a) Mechanism for the photocatalytic decomposition of NPX by co-doped TiO_2_. (b) NPX degradation efficiency with the photocatalyst in the presence and absence of various scavengers. This figure was adapted with permission from *Journal of Environmental Science and Health, Part A*, 2019, **54**, 1254–1267.^145^

Another strategy to simplify the degradation of pharmaceuticals using a catalytic system is to narrow the absorption range of TiO_2_ to the visible light region. Besides the abovementioned method, another way includes doping non-metal species in the oxygen sites of the TiO_2_ structure, creating oxygen defects, which reduce the bandgap energy of non-metal-doped TiO_2_ and lowers the energy level of its VB. As an example, a nitrogen-doped TiO_2_/SiO_2_/Fe_3_O_4_ magnetic nanocomposite (NTSF) exhibited 96.32% efficiency for the degradation of naproxen (NPX) under the optimum conditions.^145^ As predicted, nitrogen doping improves the photocatalytic activity of TiO_2_ because it reduces the bandgap energy of the composite to 2.9 eV. Thus, it can be photocatalytically active under purple light-emitting diode (LED) illumination. Also, SiO_2_ acts as an efficient agent in the nanocomposite to enhance its specific surface area, lower its bandgap and obstruct the state change of TiO_2_ from anatase to rutile. The reduction in magnetic saturation from 45.40 emu g^−1^ for Fe_3_O_4_ to 30.45 emu g^−1^ for NTFS is attributed to the non-magnetic SiO_2_ layer and heating during the calcination process. However, NTSF was reused in four consecutive cycles without any significant reduction in its efficiency. In general, as previously stated, reactive oxidative species (ROS) such as ˙OH, O_2_˙^−^, h^+^, and e^−^ promote photocatalytic reactions (Fig. 22a). Various scavenger agents were applied to evaluate the level of ROS participation in the photocatalytic reactions. The photocatalytic activity of NTSF in the degradation of NPX under the optimized conditions was investigated in the presence and absence of different scavengers (Fig. 22b). The greater the rate of degradation, the more effective role the radical plays in the degradation process. Ammonium oxalate (AO), benzoic acid (BA), *p*-benzoquinone (BQ), and K_2_Cr_2_O_7_ are the appropriate scavengers for h^+^, ˙OH, O_2_˙^−^, and e^−^, respectively. The degradation rate was considerably reduced with the addition AO, BQ, K_2_Cr_2_O_7_, and BA. Thus, it was concluded that the destruction of NPX was mainly conducted by ˙OH and h^+^ played a minor role in the degradation process.

**Fig. 23:**
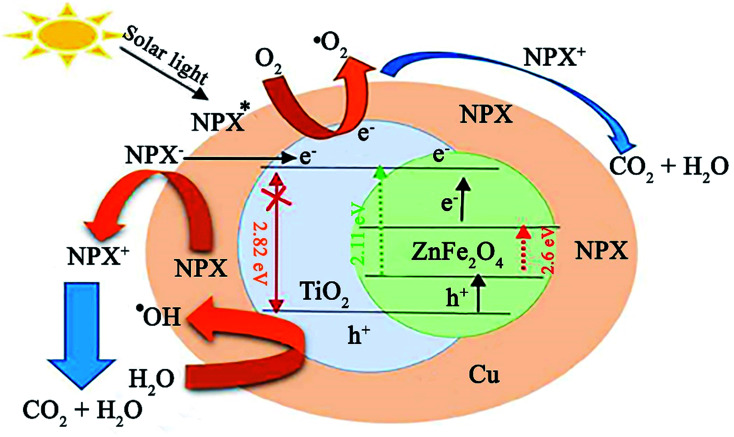
Plausible mechanism for the photocatalytic degradation of NPX under a solar light source. This figure was adapted with permission from *Journal of Cleaner Production*, 2020, **268**, 122023.^146^

In a recent study by Sayadi *et al.*, the degradation of naproxen (NPX) was conducted using ZnFe_2_O_4_@TiO_2_/Cu under solar light irradiation.^146^ The participation of ZnFe_2_O_4_ with a low bandgap of 2.11 eV in the composite photocatalyst caused it to possess a narrow bandgap of about 2.62 eV, which resulted in photocatalytic activity under visible light. Initially, to balance the total energy of the system, the electrons in the copper NPs transfer to TiO_2_ given that they have higher energy levels (Fig. 23). Simultaneously, when NPX contacts the ZnFe_2_O_4_@TiO_2_/Cu photocatalyst, the electrons in the VB of TiO_2_ and ZnFe_2_O_4_ transfer to their CB by excitation from sunlight irradiation. The fate of these stimulated electrons can be predicted in different ways. The produced electrons in the CB of TiO_2_ by solar light irradiation are transferred to Cu. In the next step, they may first transfer from the CB of ZnFe_2_O_4_ to the CB of TiO_2_ and then move to Cu.

Further, direct electron injection from the CB of ZnFe_2_O_4_ to Cu NPs is plausible. Accordingly, the ZnFe_2_O_4_@TiO_2_/Cu photocatalyst can diminish the recombination rate of excitons. The holes in the VB of ZnFe_2_O_4_ and TiO_2_ are involved in the degradation of NPX and ˙OH generation, while the photogenerated electrons react with O_2_ for the production of more O_2_˙^−^. Lastly, the resultant drugs turn into H_2_O and CO_2_ by ˙OH, O_2_˙^−^, and h^+^ radicals. This catalyst exhibited not only high removal efficiency for NPX (80.73%) but also exhibited superior reusability, where after five runs of degradation, 72.31% degradation was attained. In addition, facile recovery of this core–shell ZnFe_2_O_4_@TiO_2_/Cu structure was achieved due to its magnetic saturation of 26.45 emu g^−1^, which is comparable with that of other magnetic TiO_2_ photocatalysts.

Also, a magnetic TiO_2_–graphene oxide–Fe_3_O_4_ composite was prepared by He *et al.* for the photo-Fenton decomposition of amoxicillin.^147^ Although each structural moiety of this photocatalyst has its unique beneficial properties, an excellent synergic effect occurred after they were combined. For instance, graphene oxide (GO) with properties such as 2D carbonaceous monolayer structure, high surface area, and electrical conductivity was employed as a template for binding nanoparticles. Besides, Fe_3_O_4_ has high chemical and thermal stability compared to other oxides and it aids the simple recovery of the catalyst. According, all these parts in the TiO_2_–graphene oxide–Fe_3_O_4_ composite aimed to enhance the catalytic role, durability and separation property deficiencies of TiO_2_ by its combination with the high-conductive (GO) and high-magnetic recovery ability (Fe_3_O_4_) of its components.

**Fig. 24:**
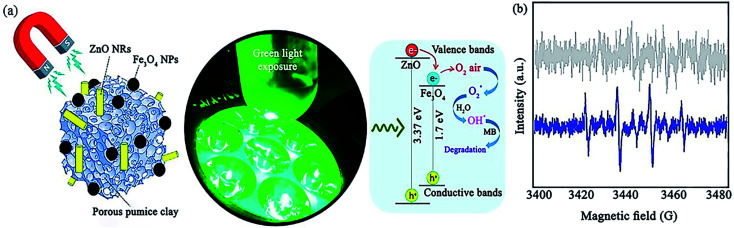
(a) Illustration of the photocatalytic mechanism for the degradation of MB with synergistic effect between green light exposure and ZnO/Fe_3_O_4_@pumice photocatalyst. (b) ESR spectra for DMPO scavenger of ZnO/Fe_3_O_4_@pumice photocatalyst at ambient temperature. This figure was adapted with permission from *Materials Research Bulletin*, 2020, **130**, 110946.^148^

ZnO is another useful photocatalyst that contributes in catalytic systems to the degradation of organic contaminants. In a recent report, Maleki *et al.* prepared a ZnO/Fe_3_O_4_@pumice photocatalyst for the degradation of methylene blue (MB) under green light irradiation with a maximum photocatalytic efficiency of 85.5% (Fig. 24a).^148^ As another strategy, the improvement in magnetic property obtained through composing magnetic pumice micro-plates and Fe_3_O_4_ NPs was proved by the ∼20 emu g^−1^ increase in the magnetic saturation of Fe_3_O_4_@pumice at ±15 000 Oe magnetic field. The simple retrievability of the photocatalyst for eight cycles was attributed to its high magnetic property. The same as-proposed mechanisms for TiO_2_ and ZnO nanorods (NRs) was stimulated when exposed to green LED light.

Regarding the resemblance of the energy level of the CB of ZnO and Fe_3_O_4_, the same number of holes produced in their CB results from the electron transfer from the VB of ZnO to the VB of Fe_3_O_4_. This procedure somehow reduces the electron–hole pair recombination. Therefore, electron accumulation in the VB of ZnO enhances the production of O_2_˙ from the O_2_ molecules in the air. Moreover, ˙OH radicals are generated from water during the oxidization process of the holes (h^+^). In this report, erythrocyte sedimentation rate (ESR) analysis was performed to screen the formation of the ˙OH radical (Fig. 24b). Accordingly, 5,5-dimethyl-1-pyrroline *N*-oxide (DMPO) as a conventional spin-trap reagent was applied. It was concluded that ˙OH radicals play a vital role in the degradation of MB compared to O_2_˙ radicals because there was no distinctive signal in the ESR spectrum when the reaction with ZnO/Fe_3_O_4_@pumice was carried out in the dark. However, the DMPO–OH adduct exhibited four quartet peaks under green-light exposure. Considering that the individual bandgap of ZnO is 3.32 to 3.37 eV, it absorbs UV radiation, which is not very favorable. Thus, the composition process with Fe_3_O_4_ owing to its bandgap of 1.7 eV leads to more effective photocatalytic degradation and efficient energy harvesting ability in comparison with bare ZnO NRs.

It should also be noted that the contribution of magnetic nano spinel ferrites to 2D graphene family nanomaterials result in preferential properties compared to their individual catalytic systems. Graphene-based nano spinel ferrites (GNSFs) are potentially cost-effective and environmentally friendly materials and possess improved physical and chemical characteristics such as restrained particle aggregation, boosted active surface area, and simple magnetic removal for the recycling process.^149,150^ These magnetic catalysts participate in both adsorptive and degradation reactions.^151^ GNSFs show incomparably better adsorption efficiency than individual graphene-based nanomaterials, *i.e.*, graphene, GO, and rGO. As a prominent example, rGO/Bi_2_Fe_4_O_9_ displayed a maximum adsorption capacity (*q*_m_) of 3.95 mg g^−1^ for bisphenol-A, which is significantly higher than that of Bi_2_Fe_4_O_9_ (0.74 mg g^−1^) and GO/Bi_2_Fe_4_O_9_ (1.72 mg g^−1^).^152^ This remarkable result is related to the enhanced surface area of rGO (∼2600 m^2^ g^−1^) and π–π stacking interactions between the benzene ring of bisphenol-A and surface functional groups of rGO. GNSFs demonstrate a high degree of light-harvesting features and a broader visible light absorption spectrum because of the participation of graphene in magnetic catalytic systems. In the optimized state, it was found that as the graphene content increases, the absorption of visible light increases.

**Fig. 25:**
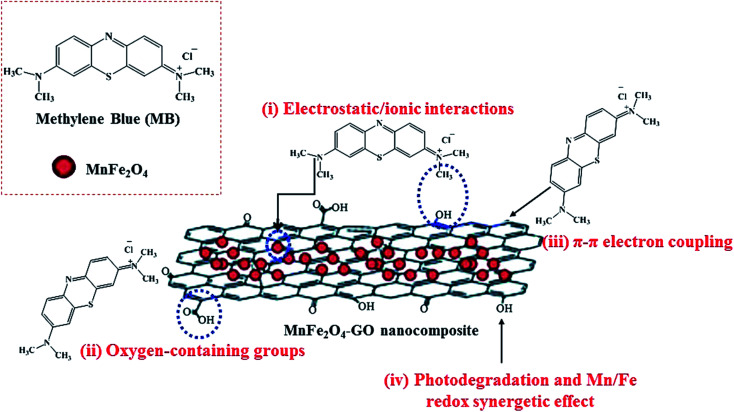
Illustration of methylene blue (MB) adsorption mechanism on GO–MnFe_2_O_4_ nanocomposites. This figure was adapted with permission from *RSC Advances*, 2018, **8**, 12376–12389.^154^

Regarding the study done by Liu *et al.*, graphene in GNSFs acts as an acceptor and mediator agent for photogenerated electrons, which impedes the recombination of e^−^–h^+^ pairs during the transition process. The role of rGO relies on its substantial π–π network, which retains the electrons, and its lower Fermi energy compared to the CB of ZnFe_2_O_4_.^153^ Reportedly, manganese ferrite/graphene oxide (MFO–GO) was applied for the adsorption of MB from an aqueous solution with a maximum adsorption capacity (*q*_m_) of about 177.3 mg g^−1^ (Fig. 25).^154^ The magnetic saturation of the MFO NPs was 20 emu g^−1^; however, the magnetic saturation for MFO–GO was reduced with an increase in the GO content from 12.2 to 3.2 emu g^−1^, altering the GO content from 10% to 50%, respectively. Also, MFO–GO showed excellent reusability after five cycles. From a mechanistic aspect, there are four possibilities for the removal of MB dye in neutral solution. The first is associated with electrostatic/ionic interactions between the MB molecules with a positive charge and surface OH groups of GO and MFO. The second reason is assigned to the abundant active binding sites of the basal planes and GO edges, including carboxyl (–COOH), epoxy (C–O), and hydroxyl (–OH) oxygen-containing functional groups, which act as major adsorption sites for MB. The third factor is related to the π–π interactions between the C=C double bonds of MB and π electrons of the benzene ring with the π electrons on the surface of GO. The forth item to be considered is associated with the synergistic effect of adsorption and photocatalysis, which result in the degradation of MB. As other effective items, the Mn/Fe synergistic mechanism in MFO catalysts for the decomposition of MB should be considered. As stated above, by considering the critical role of GO, more oxygen-containing functional groups can be obtained by increasing the content of GO, which agrees with the results of enhanced adsorption activity. Thus, the adjustment of the GO content and MFO NPs in composite systems can ultimately optimize the MB adsorption mechanism.

**Fig. 26:**
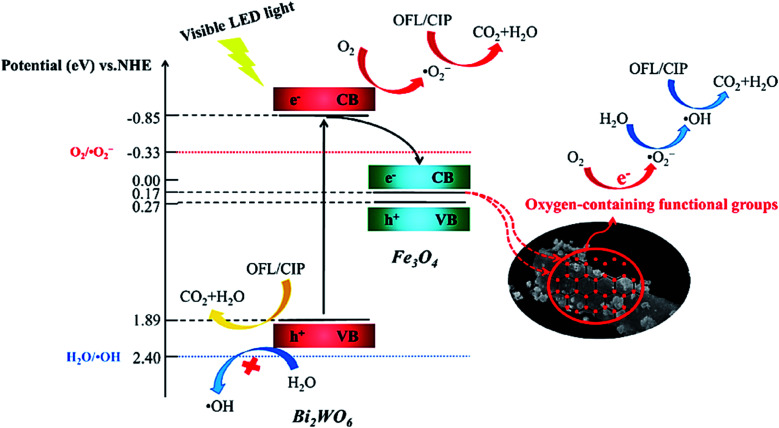
Plausible mechanism for photocatalytic degradation of OFL and CIP by Bi_2_WO_6_/Fe_3_O_4_/BC under visible LED light irradiation. This figure was adapted with permission from *Science of The Total Environment*, 2021, **764**, 142879.^161^

In addition to the mentioned magnetic photocatalytic systems, magnetic biochar (MBC) from the biochar composite family not only exhibits the advantageous features of biochar (BC) but can also be magnetically separated from the reaction flask.^155^ Moreover, due to the good contribution and dispersal of photocatalysts on the MBC support, their recovery and performance are enhanced, and their aggregation will be effectively inhibited.^156^ MBC-catalyzed decomposition systems competently decrease the organic content of pollutants, which can be traced by total organic carbon (TOC) analysis.^157,158^ Additionally, the degraded contaminants may be more noxious and toxic than the individual pollutants given that the mineralization of organic pollutants does not completely occur.^159^ Fortunately, researchers have proven the detoxification effect of MBC-catalyzed degradation systems, which is an excellent feature compared to other degradation systems.^160^ In a recent study conducted by Xie *et al.*, a Bi_2_WO_6_/Fe_3_O_4_/BC photocatalyst was synthesized *via* a hydrothermal method to degrade ofloxacin (OFL) and ciprofloxacin (CIP) upon exposure to a visible LED.^161^ The intermediate products of OFL and CIP exhibited less toxicity against *Escherichia coli* bacteria than the original pharmaceutical contaminants. As depicted in Fig. 26, by irradiating Bi_2_WO_6_ with a visible LED light, e^−^ transfers from its VB (1.89 eV) to CB (−0.85 eV). Given that the reduction potential of O_2_/O_2_˙^−^ (−0.33 eV *vs.* NHE) is lower than the CB potential of Bi_2_WO_6_, oxygen molecules can be reduced to form O_2_˙^−^ by the electrons in the CB. However, the VB potential of Bi_2_WO_6_ is not positive enough to generate ˙OH (+2.40 eV *vs.* NHE). In this regard, the ˙OH production proceeds *via* the oxygen-containing functional groups of BC. The cascade electron transition first transfers e^−^ to O_2_ to form O_2_˙^−^. Subsequently, H_2_O_2_ is produced under the actions of the electron. Finally, the produced H_2_O_2_ is degraded to form ˙OH. As mentioned, the main and vital factors to produce ˙OH in these catalytic degradation systems are the oxygen-containing functional groups on BC. Specifically, the h^+^, O_2_˙^−^, and ˙OH radicals are involved in both the degradation and mineralization of OFL and CIP. Table 2 presents some examples of magnetic catalytic systems applied for degradation of the pharmaceutical ingredients with high removal efficiency.

**Table 2:** Brief information of some magnetic catalytic systems applied for the degradation of organic compounds

Entry	Catalyst	Function	Pollutant	Pollutant removal (%)	Ref.
1	Fe_3_O_4_@BC^a^	Fenton-like degradation	Metronidazole (MNZ)	100	162
2	γ-Fe_2_O_3_@BC	Degradation	Tetracycline (TC)	82.24	163
3	rGO^b^/gadolinium doped ZnFe_2_O_4_ (GZFG)	Adsorption	Levofloxacin (LVX)	86	164
4	Carbon-bridge-modified MLD/CN/Fe_3_O_4_^c^	Fenton-like degradation	Tetracycline (TC)	95.8	165
5	CMCS@Fe_3_O_4_^d^	Adsorption	Doxorubicin hydrochloride (DOX)	85.46	166
6	MnFe_2_O_4_/Bi_2_MoO_6_/PPy^e^	Adsorption	Ketoprofen (KET) and indomethacin (IDM)	87.03, 86.24	167
7	SMF^f^	Photocatalytic degradation	Diclofenac (2-(2-(2,6-dichlorophenylamino)phenyl) acetic acid) (DCF)	99	168
8	CuFe_2_O_4_/Bi_2_O_3_	Degradation	Lomefloxacin (LOM)	77.19	169
9	MoS_2_/CuFe_2_O_4_	Degradation	Fluoxetine	97.7	170
10	Fe_3_O_4_/g-C_3_N_4_	Photocatalytic degradation	Tetracycline (TC)	99.8	171
11	Mn_2_O_3_–Fe_3_O_4_@BC	Degradation	Naphthalene	77.1	172
12	Ag–CuFe_2_O_4_@WO_3_	Photocatalytic degradation	Gemfibrozil (GEM) and Tamoxifen (TAM)	81, 83	173
13	Magnetite supported on multi-walled carbon nanotubes	Catalytic wet peroxide oxidation	Diclofenac (DCF) and naproxen (NAP)	54, 19	174
14	Ag_3_PO_4_/rGO/CoFe_2_O_4_	Adsorption	Levofloxacin (LVF)	90.7	175
15	rGO/NiFe_2_O_4_	Photocatalytic degradation	MB, MO, RhB	99.1, 47.1, 82.2	176
16	Bi_2_O_2_CO_3_–CoFe_2_O_4_@BC	Photocatalytic degradation	Paraquat (PQT)	99.3	177
17	CoFe_2_O_4_ NPs	Photocatalytic degradation	Atenolol (ATL)	90	178
18	Iron oxide/cellulose	Adsorption	Ciprofloxacin (CIP)	92.01	179
19	ZnO/Fe_2_O_3_	Photocatalytic degradation	Sulfamethoxazole (SMX)	95.2	180
20	Fe^0^@BC	Fenton-like degradation	Trichloroethylene (TCE)	98.9	181

a Biochar.

b Reduced graphene oxide.

c Carbon-bridge-modified malonamide (MLD)/g-C_3_N_4_ (CN)/Fe_3_O_4_.

d Carboxymethyl cassava starch (CMCS)-functionalized Fe_3_O_4_.

e Polypyrrole.

f Sn_0.15_Mn_0.85_Fe_2_O_4_.

## 4. Application of micro and nanoscale magnetic catalytic systems in the synthesis of pharmaceutical compounds

**Fig. 27:**
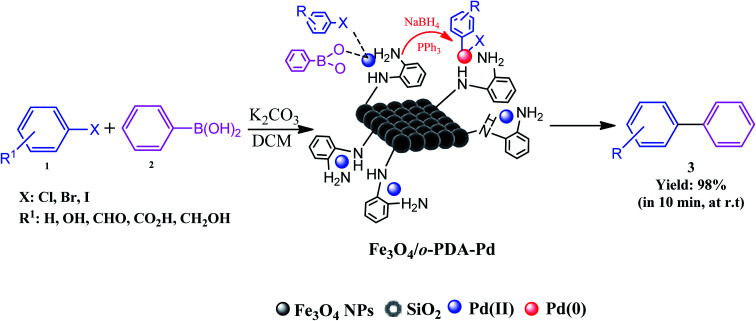
Schematic of Suzuki–Miyaura cross-coupling reaction conducted using magnetic Fe_3_O_4_/*o*-PDA–Pd nanocatalyst at room temperature (r.t). This figure was adapted with permission from *Journal of Physics and Chemistry of Solids*, 2020, **136**, 109200.^17^

In recent decades, the use of catalytic systems and their functionalities have attracted great attention due to their benefits in pharmaceutical synthesis for scaling up production. However, there are numerous issues in the field of catalysis. These issues include environmental impact, process safety, mass and heat transfer restrictions, and optimizing reaction conditions, *i.e.*, temperature, pressure, time, solvents, scaled-up procedures, reusability and stability, catalyst deactivation, and recovery. Among the catalytic systems, heterogeneous and magnetic catalysts meet the needs for convenient catalyst recovery from the reaction flask with only an external magnet. As a brilliant example, the Fe_3_O_4_/*o*-PDA–Pd nanocatalyst developed by Maleki *et al.* was applied in the Suzuki–Miyaura coupling reaction, in which biphenyls were prepared from aryl halides and phenylboronic acids, as shown in Fig. 27.^17^

**Fig. 28:**
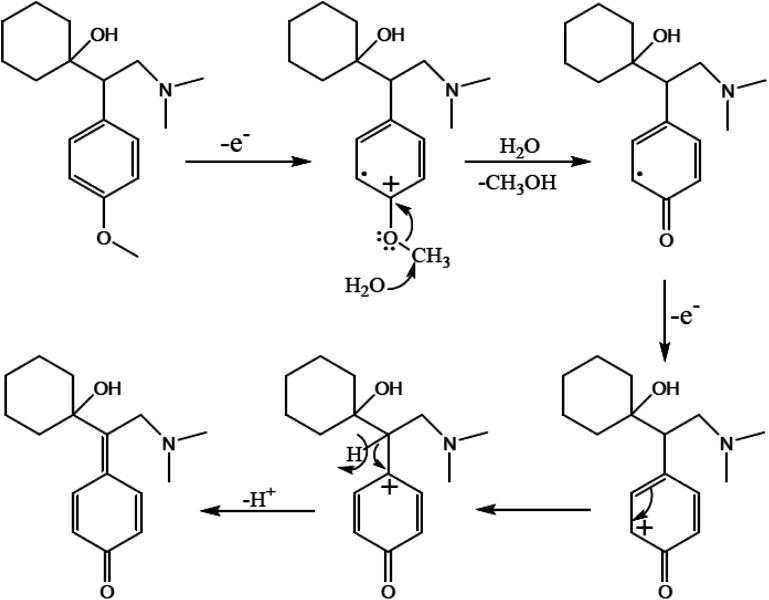
Proposed mechanism for the electrooxidation of venlafaxine at the surface of Fe_3_O_4_@nano-cellulose/Cu nanocomposite/GSPE. This figure was adapted with permission from *Industrial & Engineering Chemistry Research*, 2020, **59**, 4219–4228.^41^

The active pharmaceutical biphenyl compounds resulting from this reaction, including losartan, flurbiprofen, and tarenflurbil, are used as resources to treat hypertension and diabetic nephropathy,^182^ signs of osteoarthritis and rheumatoid arthritis,^183^ and Alzheimer's disease and prostate cancer,^184^ respectively. The surface functionalization through covalent bonding occurs between the amine functional group of PDA and chloropropyl carbons of silane, which are bonded to chlorine. Eventually, the unpaired electron pairs in the amines interact with the empty orbitals in Pd. Due to the reaction mechanism, the electron interactions among the three main components involving the oxygen atoms of aryl halides and phenylboronic acid and divalent palladium ions lead to the final covalent binding. Subsequently, under basic conditions, Pd(ii) was subjected to reduction to Pd(0) by sodium borohydride reducing agent. The reproducibility tests indicated ten times recycling for this catalyst without any reduction in its catalytic activity. It should be highlighted that by applying this catalyst in the Suzuki–Miyaura coupling reaction, approximately 98% yield was achieved in 10 min. In another study, Beitollahi *et al.* developed an environmentally friendly and facile method for the synthesis of an Fe_3_O_4_@cellulose nanocrystal/Cu nanocomposite (Fe_3_O_4_@CNC/Cu). Henceforth, a graphite screen-printed electrode (GSPE) modified with Fe_3_O_4_@CNC/Cu was applied as a sensor for the electrochemical oxidation of venlafaxine. Fig. 28 displays the possible electrooxidation mechanisms.^41^

**Fig. 29:**
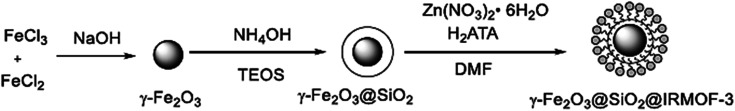
Schematic showing the preparation of the γ-Fe_2_O_3_@SiO_2_/IRMOF-3 catalyst. This figure was adapted with a permission from *Journal of Catalysis*, 2020, **387**, 39–46.^188^

**Fig. 30:**
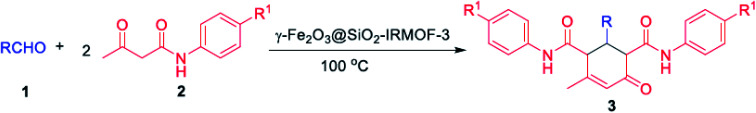
Synthesis of cyclohexenone derivatives using γ-Fe_2_O_3_@SiO_2_/IRMOF-3 catalyst. This figure was adapted with permission from *Journal of Catalysis*, 2020, **387**, 39–46.^188^

Mo *et al.* developed a magnetic recyclable metal–organic framework catalyst. They synthesized cyclohexenone derivatives under solvent-free conditions. Cyclic enone derivatives are acknowledged to be precious intermediates in pharmaceuticals and natural products. Cyclohexanone units containing C=O and C=C groups in their structure exist in many synthetic and medicinally natural products such as the fungus *A. flavus* YIM DT 10012, and *Trachyspermum roxburghianum*. Additionally, they can be applied in drugs, pesticides, and polymers.^185–187^ As depicted in Fig. 29 and 30, Zhang *et al.* researched the catalytic solvent-free synthesis of cyclohexanone derivatives through aldehyde and acetoacetanilide condensation reactions utilizing the heterogeneous γ-Fe_2_O_3_@SiO_2_/IRMOF-3 MOF catalyst. The catalyst was easily separated with an external magnet in this environmentally benign approach and retrieved several times without any changes in its catalytic activity.^188^

**Fig. 31:**
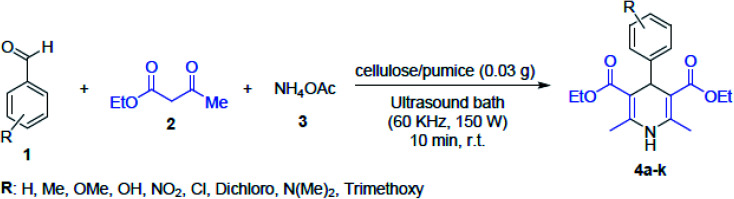
Synthetic pathway for 1,4-dihydropyridine derivatives using cellulose/pumice nanocomposite catalyst *via* sonication at room temperature (r.t). This figure was adapted with permission from *Solid State Sciences*, 2020, **101**, 106141.^189^

Maleki *et al.* introduced magnetic pumice in a novel and well-designed magnetic composite, which led to the effortless recycle of the catalyst from the reaction mixture. In this work, palladium nanoparticles as the main catalytic active sites were well-distributed on the VPMP@CLS structure. For the Suzuki–Miyaura cross-coupling catalytic reactions, as a heterogeneous catalytic system, the Pd^2+^ nanoparticles were reduced to Pd- and biphenyl pharmaceutical derivatives were produced. This product has high importance in pharmaceutical compounds. It can be applied in cancer therapy and treatment of arteriosclerosis, osteolytic disorders, and ophthalmic disorders, and used as integrin antagonists.^30^ In another study by Maleki *et al.*, a pumice magnetic volcanic rock and cellulose matrix nanocomposite was synthesized. This heterogeneous biodegradable nanocomposite was employed in the synthesis of 1,4-dihydropyridine derivatives, and the reaction outline is presented in Fig. 31. Given that 1,4-dihydropyridine derivatives have a wide variety of applications in antihypertensive and anticancer drugs, there synthesis procedures require significant consideration.^189^

**Fig. 32:**
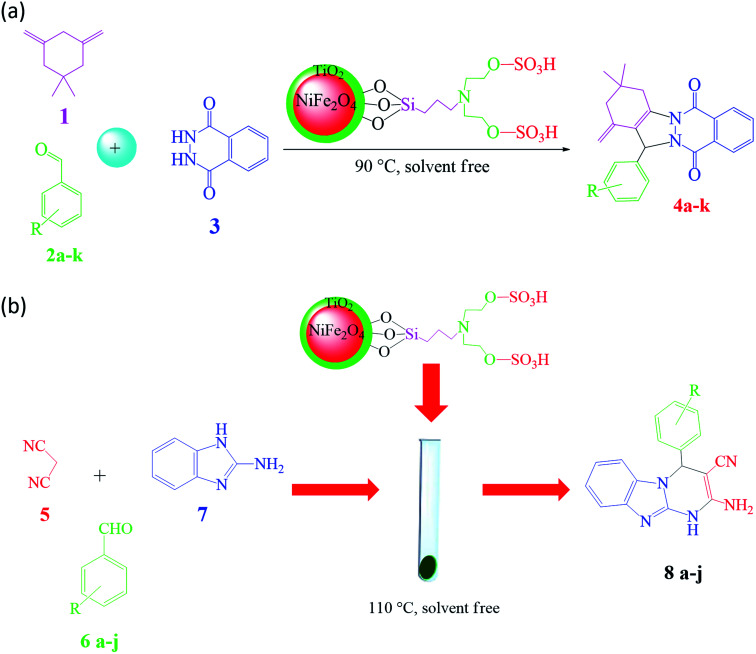
(a) Schematic of the synthetic route for 2*H*-indazolo[2,1-*b*]phthalazine-triones. This figure was adapted with permission from *Chem. Sel*., 2019, 4, 17–23.^71^ (b) Schematic of the synthetic approach for benzo[4,5]imidazo[1,2-*a*]pyrimidines. This figure was adapted with permission from *Chem. Sel.*, 2019, **4**, 17–23.^71^

Based on the advantages of heterogeneous hybrid magnetic nanocatalysts, organo-sulfonic acid tags attached to magnetic titania-coated NiFe_2_O_4_ nanoparticles were employed to form nano-NiFe_2_O_4_@TiO_2_–SiO_2_–Pr–DEA–OSO_3_H nanocatalysts to advance the green synthesis process.^71^ In this regard, the prepared hybrid nanocatalyst was utilized in the synthesis of pharmaceutical components such as 2*H*-indazolo[2,1-*b*]phthalazine-triones (Fig. 32a) and benzo[4,5]imidazo[1,2-*a*]pyrimidine derivatives through multicomponent reactions (MCRs) under moderate and green reaction conditions (Fig. 32b). In addition to good catalytic reusability (8 times), for the synthesis of 2*H*-indazolo[2,1-*b*]phthalazine-triones, a high reaction yield (97%) in just 5 min at 90 °C was obtained using only 20 g of the catalyst in a one-pot, three-component, solvent-free reaction, which is an accomplishment compared to the time taken when the reaction was performed with neat NiFe_2_O_4_. Moreover, for the synthesis of benzo[4,5]imidazo[1,2-*a*]pyrimidine derivatives at 110 °C under solvent-free conditions, 95% yield was obtained. Significantly, phthalazine-trione derivatives are well-known heterocycles used in the pharmaceutical and biological fields including vaso-relaxants,^190^ antifungal,^191^ antimicrobial,^192^ anti-cancer,^193^ and anti-inflammatory agents.^194^ Besides, benzo[4,5]imidazo[1,2-*a*]pyrimidines are biologically related fused pyrimidine derivatives, which demonstrate highlighted therapeutic and biological characteristics such as antimicrobial,^195^ anti-inflammatory,^196^ protein kinase inhibitor,^197^ and anticancer.^198^

**Fig. 33:**
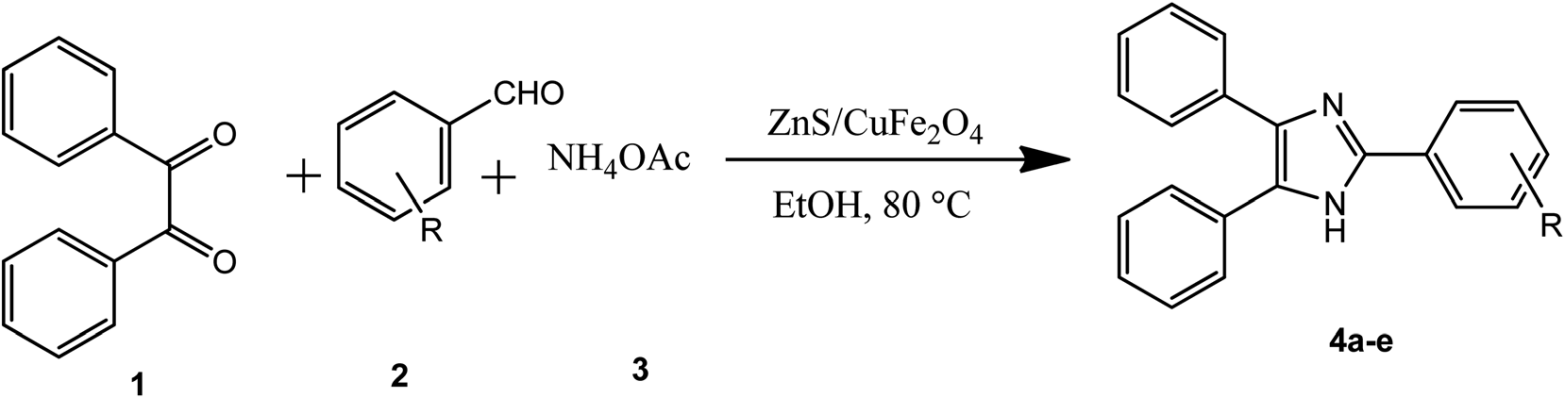
Schematic showing the synthesis of 2,4,5-triaryl-1*H*-imidazole derivatives. This figure was adapted with permission from *Multidisciplinary Digital Publishing Institute Proceedings*, 2019, pp. 44 (DOI: 10.3390/ecsoc-23-06654).^70^

In this report, a hybrid magnetic nanocomposite, ZnS/CuFe_2_O_4_, was applied as a heterogeneous catalyst to synthesize 2,4,5-triaryl-1*H*-imidazole derivatives through the one-pot condensation of various aromatic aldehydes, benzyl and ammonium acetate, as depicted in Fig. 33. The imidazole nucleus acts as the leading scaffold to form significant biological active molecules with antibacterial, antifungal, anti-inflammation, anticancer, antiviral, anti-diabetic, anti-allergic, analgesic and herbicidal functionalities.^199,200^ Some aromatic aldehydes were used in the synthesis of 2,4,5-triaryl-1*H*-imidazole derivatives. The aromatic aldehydes produced the desirable corresponding products in 82–92% yield due to their electron withdrawing and donating substituents.

**Fig. 34:**
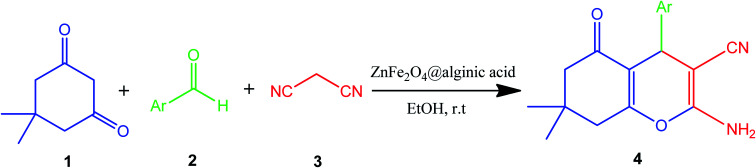
Synthesis of 2-amino-3-cyano-4*H*-pyran derivatives at room temperature (r.t). This figure was adapted with permission from: *Polyhedron*, 2019, **171**, 193–202.^75^

According to Maleki's report, 2-amino-3-cyano-4*H*-pyran derivatives were synthesized using the ZnFe_2_O_4_@alginic acid heterogeneous nanocatalyst through the condensation reaction of dimedone (1), aromatic aldehydes (2) and malononitrile (3) in ethanol at room temperature (Fig. 34).

**Fig. 35:**
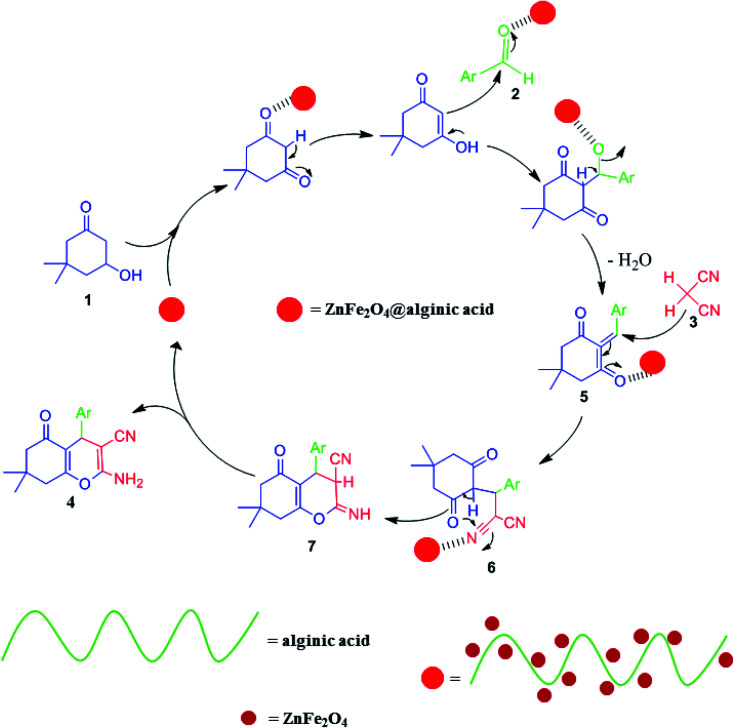
Suggested mechanism for the synthesis of 2-amino-3-cyano-4*H*-pyran derivatives. This figure was adapted with permission from *Polyhedron*, 2019, **171**, 193–202.^75^

Precisely, due to the synergistic effect of the active sites of alginic acid (several hydroxyl and carboxylic acid groups in its structure) and Lewis acid centers of ZnFe_2_O_4_, the hybrid catalyst demonstrated a remarkable yield of 93% in 10 min under the optimized conditions compared to its individual components. In the first stage, the heterogeneous nanocatalyst activates the carbonyl groups of dimedone and aldehyde in two paths from a mechanistic view. The formation of a hydrogen bond results from the hydroxyl or carboxylic acid active groups in alginic acid as the organic constituent of the catalyst or Lewis acid centers of ZnFe_2_O_4_ as the inorganic part. Then, the activated aldehyde and dimedone are exposed to catalytic Knoevenagel condensation to form molecule (5). Afterwards, the malononitrile as a C–H acid attacks the intermediate through Michael addition. In the next step, an intramolecular cyclization happens for molecule (5) to form intermediate (7). Eventually, the product formation is implemented by tautomerization of intermediate (7). The proposed mechanism is shown in Fig. 35.

The high reaction yield in the range of 83–95% for ten 2-amino-3-cyano-4*H*-pyran derivatives should be highlighted to indicate the other strengths of this study. Also, a convenient purification procedure with high stability and reusability for five sequential catalytic cycles was carried out. Notably, the 4*H*-pyran family and their derivatives are the main parts in the production of natural and chemical molecules. They exhibit extensive favorable pharmaceutical applications such as anticancer, diuretic, spasmolytic, antibacterial, anti-HIV, antimalarial, anti-inflammatory, antihyperglycemic and dyslipidemia activities.^201,202^ Besides, they have demonstrated therapeutic effects in some neurodegenerative disorders such as Parkinson's disease and Alzheimer's disease.^203^ Also, a magnetic MOF catalyst, NiFe_2_O_4_@MOF-5, was applied as a heterogeneous catalyst to synthesize 2-substituted alkyl and aryl(indolyl) kojic acid derivatives through the solvent-free, one-pot, three-component reaction of aldehyde, indole, and kojic acid.^83^Table 3 lists some of the magnetic catalytic systems that have been suitably utilized for the synthesis of pharmaceutical compounds under mild reaction conditions.

**Table 3:** Brief information of some magnetic catalytic systems applied in the synthesis of pharmaceutical compounds

Entry	Catalyst	Function	Yield^a^ (%)	Ref.
1	Nano-Fe_3_O_4_@(HSO_4_)_2_	Synthesis of 2-amino-3-cyanopyridines and hexahydroquinoline derivatives	53–89.4, 67.97–98.4	204
2	WO_3_ZnO/Fe_3_O_4_	Synthesis of 2-substituted benzimidazole derivatives	88–98	205
3	Fe_3_O_4_@KCC-1^b^–npr–NH_2_	Synthesis of sulfonamide derivatives	85–97	206
4	CoFe_2_O_4_ MNPs	Synthesis of 2,4,5-trisubstituted imidazoles	83–93	207
5	Fe_3_O_4_@CS^c^–Co	Synthesis of aryl nitriles and biaryls	60–85, 62–91	208
6	MPIL^d^	Synthesis of 2′-aminobenzothiazolomethylnaphthols and amidoalkyl naphthols	87–96, 75–94	209
7	Pd@Fe_3_O_4_/AMOCAA^e^	Suzuki and Sonogashira cross-coupling reactions	60–100, 79–96	210
8	γ-Fe_2_O_3_@cellulose-OSO_3_H	Synthesis of 2,4-dihydropyrano[2,3-*c*]pyrazole and spiro[indoline-3,4′-pyrano[2,3-c]pyrazole derivatives	84–99, 89–95	211
9	Cu NPs@Fe_3_O_4_-chitosan	Synthesis of amino- and *N*-sulfonyl tetrazoles	81–90	212
10	Pd NPs @Fe_3_O_4_/lignin/chitosan	Cyanation of aryl halides and coupling reactions	60–97, 70–97	213
11	Iron oxide@PMO^f^-PrSO_3_H	Synthesis of imidazopyrimidine derivatives	88–96	214
12	Fe_3_O_4_@SiO_2_@(CH_2_)_3_–urea–benzimidazole sulfonic acid	Synthesis of 2-amino-3-cyano pyridine derivatives	70–92	215
13	γ-Fe_2_O_3_/Cu@cellulose	Synthesis of 1,4-dihydropyridine derivative and polyhydroquinolines	80–93, 80–98	216
14	Cu NPs/MZN^g^	Synthesis of 1,2,3-triazoles	90–98	217
15	Fe_3_O_4_@SiO_2_@Si(CH_2_)_3_Cl with morpholine tags	Synthesis of hexahydroquinolines and 2-amino-4,6-diphenylnicotinonitriles	78–87, 81–95	218
16	SrFeGO^h^	Synthesis of β-enamino ketones	80–98	219
17	Fe_3_O_4_@NFC^i^-Im^j^Saloph^k^Cu	Synthesis of 1,4-disubstituted 1,2,3-triazoles	75–97	220
18	Pd–γ-Fe_2_O_3_-2-ATP^l^–TEG–MME^m^	C–C cross-coupling reactions including cyanation reaction of iodobenzene with K_4_[Fe(CN)_6_]·3H_2_O, fluoride-free Hiyama reaction of halobenzenes with triethoxyphenylsilane, and Suzuki reaction of various aryl halides with phenylboronic acid	52–99, 51–96, 80–98	221
19	MMNPs^n^	Synthesis of thiazolidinones	93–98	222
20	[Fe_3_O_4_@GON–(pyridin-4-amine)]^o^	Synthesis of 4*H*-chromenes and dihydropyrano[2,3-*c*]pyrazole derivatives	10–98, 30–98	223

a Isolated yield.

b Fibrous nano-silica.

c Chitosan.

d Magnetic phosphonium ionic liquid.

e 2-(7-Amino-4-methyl-2-oxo-2*H*-chromen-3-yl) acetic acid.

f Highly-ordered periodic mesoporous organosilica.

g Magnetic zeolite nanocomposite.

h Graphene oxide.

i Nano-fibrillated cellulose.

j Imidazole.

k Salophen.

l 2-Aminothiophenol.

m Triethylenglycol monomethyl ether.

n Copper/Schiff base complex immobilized on amine-functionalized silica mesoporous magnetic nanoparticles.

o Fe_3_O_4_-magnetized *N*-pyridin-4-amine-functionalized graphene oxide.

## 5. Magnetization in micro and nanoscale materials

**Fig. 36:**
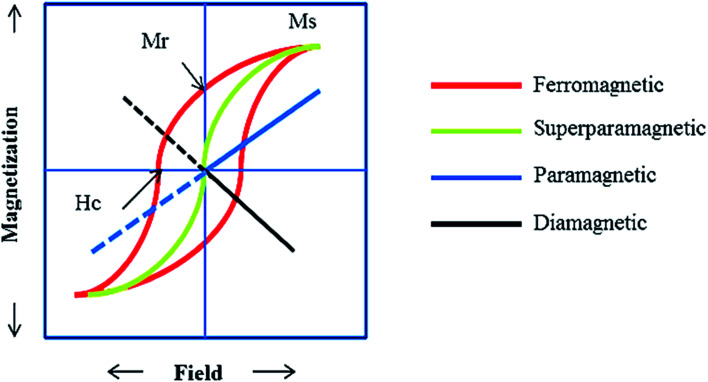
Magnetic behavior under the influence of an applied field. This figure was adapted with permission from *Int. J. Mol. Sci.*, 2013, **14**, 15977–16009.^230^

Magnetic nanoparticles (MNPs) were studied over 50 years ago just based on the specific difference in their high surface-to-volume ratio to bulk materials.^224^ Since then, impressive signs of the synthesis progress of MNPs have been seen regarding their sizes, shapes, compositions, and core–shell structures.^225^ The sensitivity and efficiency of MNPs are directly related to their higher saturation magnetization (maximum magnetization possible). The effects of geometry and shape such as composition variation are pivotal factors in enhancing their magnetic properties.^226^ Core–shell MNPs are dependent on the ligand and the surface interactions of nanoparticles, the effect of the relative thickness of their shell, and size of the coated nanoparticles.^227^ Briefly, the size, shape, composition and core–shell design in an appropriate synthesis are fundamentals of nano magnetization, which play vital roles in tuning the magnetic properties. Also, the intrinsic and extrinsic magnetic properties of materials should be considered. Atomic and molecular structure define the magnetic dipole and net magnetization to determine the diamagnetic, paramagnetic, ferromagnetic, ferrimagnetic, and antiferromagnetic properties.^228,229^ As can be seen in Fig. 36, the behavior of a material is entirely different due to the adjustment and response of its magnetic dipoles in the presence and absence of an external magnetic field.^230^ Superparamagnetic property has special importance because of drug delivery and MRI. Magnetic particles are referred to as superparamagnetic due to their magnetic properties upon the removal of an external field.

**Fig. 37:**
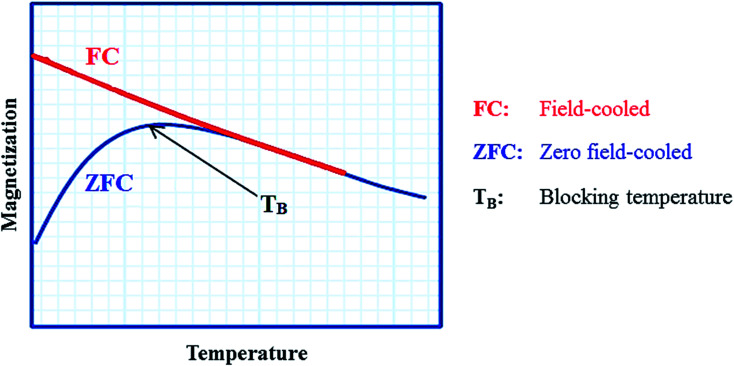
Effect of temperature. This figure was adapted with permission from *Int. J. Mol. Sci.*, 2013, **14**, 15977–16009.^230^

The effect of temperature changes the behavior of ferromagnetic and ferrimagnetic nanoparticles if they are considered in the superparamagnetic category (Fig. 37). This is the case when the measuring time is longer than the relaxation time; however, when the measurement time is shorter than the relaxation time, the nanoparticles are in a “blocked” (ferromagnetic) regime.^230,231^

**Fig. 38:**
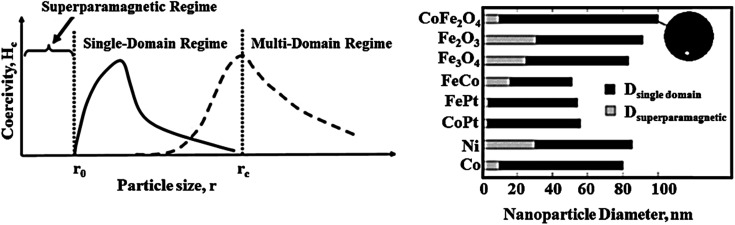
Particle size and its effect on magnetization. This figure was adapted with permission from *Int. J. Mol. Sci.*, 2013, **14**, 15977–16009.^230^

1 *T*_B_ = *KV*/25*k*_B_ = *K*(4*τr*_0_^3^/3)/25*k*_B_

where *k*_B_ is the Boltzmann constant, *K* is an anisotropy constant, and *V* is the volume of one MNP. The magnetic anisotropy energy is the energy that retains the magnetic moment in a particular orientation. Eqn (1) shows that the blocking temperature (TB) increases quickly with particle size, which is pertinent for only small MNPs. The multi-domain, single-domain and superparamagnetic regimes exhibit more magnetic behavior if the MNP size becomes smaller.^230,232^ If the MNP is synthesized below the critical volume/size, it tends to be a single magnetic domain structure, whereas if it is bigger than the critical volume, it tends to be multi-domain, and the optimized synthesized MNP tends to the superparamagnetic regime (Fig. 38).^230^ The domain wall thickness depends on the exchange energy (which is the energy required to retain the spins parallel and is low when there is a thick wall), magnetization, and anisotropy of the nanoparticle.^233^

We discussed the size of nanoparticles in the previous subsection regarding the relationship between nanoparticle size and magnetic properties, although there is another pivotal factor. The shape of nanoparticles plays a vital role in the differentiation of their magnetic properties. Nanorods, nanodiscs, nanowires, nanoflowers and tetrapods are some different shapes that exhibit varying properties. CoFe_2_O_4_ (cube and sphere) differs in coercivity, γ Fe_2_O_3_ (cube and sphere) differs in coercivity and higher TB, FePt (cube, octapod and cuboctahedron) differs in coercivity and TB, and Fe_3_O_4_ (cube and sphere) differs in TB.^234^ Researchers relate these differences to the less surface pinning according to fewer missing coordinating oxygen atoms.^235^ In addition to the unique morphology-generated gradient for the magnetic field, a higher surface-area-to-volume ratio is related to this variation, where more protons are present close to the magnetic field. However, no conclusion favours a particular shape, although MNPs with flat surfaces demonstrate promising biomedical applications to eliminate large aggregates in the body rather than gathering in the tumor.^236^

**Fig. 39:**
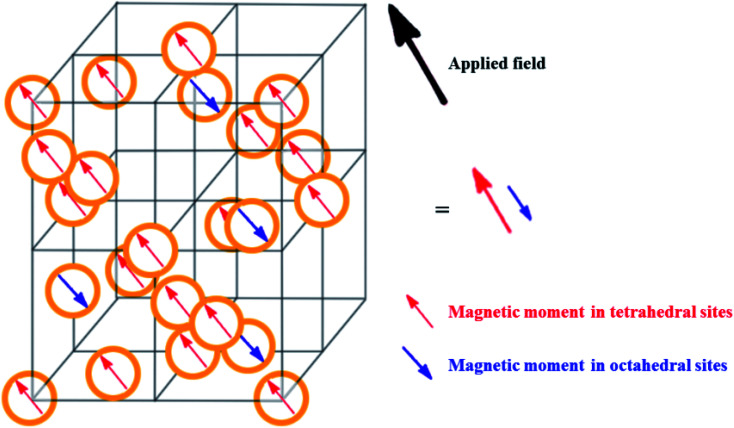
Spinel structure of ferrites, tetrahedral and octahedral. This figure was adapted with permission from *Int. J. Mol. Sci.*, 2013, **14**, 15977–16009.^230^

Finally, we discuss the composition of MNPs for the specific magnetic characteristic determination that defines their main behavior. Precursor concentration, synthesis route, and the dopant character specifically impact the magnetic properties of composites.^237^ Varying the precursor ratio affect the coercivity and *M*_s_ (emu g^−1^) and changes the magnetic properties. The cation distribution in the octahedral and tetrahedral sites, in addition to the nature of the cation itself, defines their proper replacement by dopants (Fig. 39). Cationic exchange is an applicable method to produce various structures for specific applications.^230,238^

## 6. Reusability of micro and nanoscale magnetic catalytic systems

**Fig. 40:**
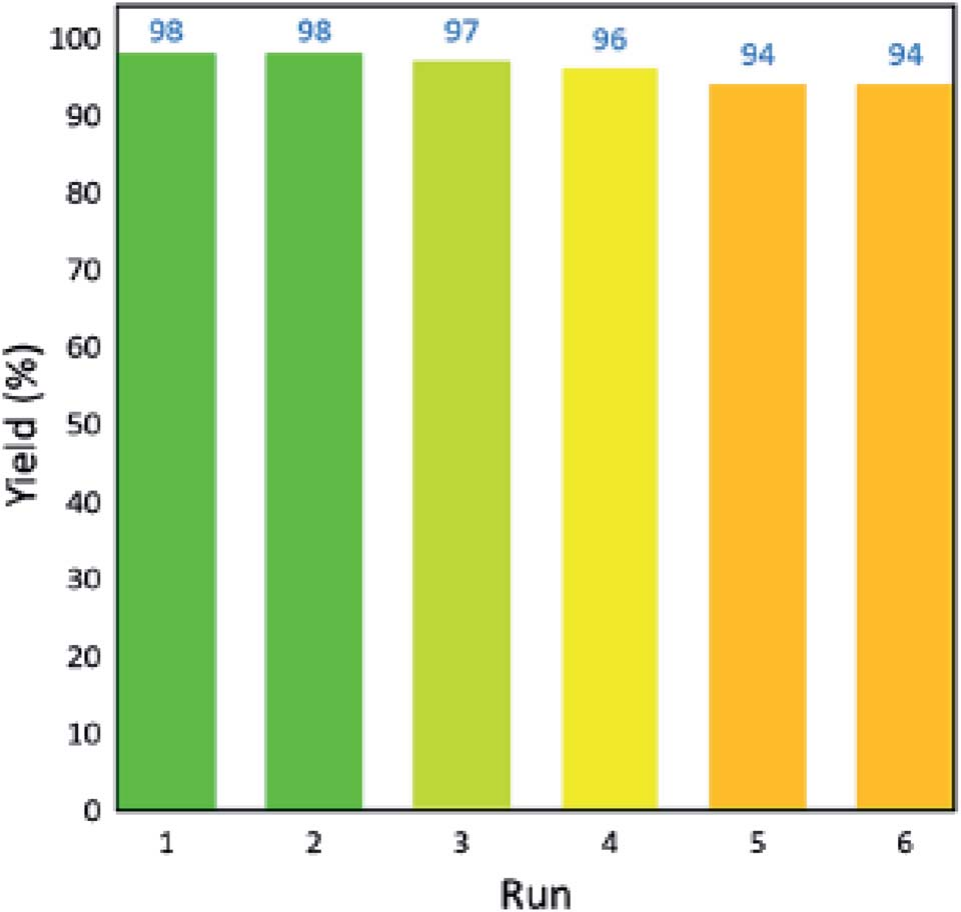
Reusability diagram of the natural igneous pumice as a catalyst. This figure was adapted with permission from Research on *Chemical Intermediates*, 2020, **46**, 4113–4128.^244^

One of the most significant parameters of catalytic systems is their reusability, which affects their commercial applicability, suitability, and sustainability. Also, the reusability of catalysts plays a vital role in the catalytic oxidation process and impacts the cost of operation and catalyst separation in various applications.^239^ Catalysts are introduced to optimize successive reaction cycles after their recovery, rinsing, and drying to investigate their reusability.^159,240–242^ Magnetic catalysts have higher reusability due to their facile workup using only a magnet. In this case, due to the lower metal leaching, the catalytic efficiency increases.^243^ Maleki *et al.* introduced a volcanic-based mesoporous magnetic catalytic system, pumice, with excellent reusability (six consecutive times), which authenticates the beneficial industrial applications of this natural-based catalyst, as shown in Fig. 40.^244^

It was proved that the experimental procedure increased the reusability of the catalyst. For example, the regeneration of magnetic nanoscale zero-valent iron (nZVI)@Ti_3_C_2_-based MXene nanosheets by treatment with dilute HCl demonstrated an optimal method to enhance their reusability. The surface inactivation of the nZVI particles (nZVIPs) was caused by the dense iron-based oxide layer covering the active sites, resulting in poor reusability.^245^ A slight loss in activity is mainly attributed initially to the leaching of metal ions from the catalyst during the reaction, recovery, and washing steps, resulting in a loss of active sites. Secondly, some residual by-products on the catalysts may cause the active sites to be obstructed, reducing the degradation yield of organic pollutants. Moreover, in highly porous catalysts, pore blockage during the reaction is highly probable, leading to a decrease in catalytic activity.^172,246^Table 4 displays some magnetic catalytic systems with improved reusability.

**Table 4:** Brief information of some magnetic catalytic systems with improved reusability and structural stability

Entry	Catalyst	Improved properties	Ref.
1	Magnetic graphene oxide	Excellent catalytic efficiency, operational durability, and recyclability	258
2	Hydrophobic virus-like organosilica nanoparticles	Improved pH and thermal resistance, high tolerance to organic solvents and long-term storage stability	259
3	Chitosan-cross-linked magnetic nanoparticles	Superior separation and biocatalytic properties	260
		Excellent storage stability and reusability	
4	Barium ferrite magnetic microparticles	Enhanced thermostability and recyclability for three successive batches	261
5	Glutathione-coated gold magnetic nanoparticles	Enhanced storage and reusability stability. Immobilized biocatalyst showed good stability after ten repeated cycles	262
6	Ionic liquid-modified magnetic chitosan composites	Enhanced thermal stability and reusability after ten cycles of reuse	263

Generally, in the scope of catalyst reusability, the chemical, physical, and mechanical stability of a catalysts should be monitored to accommodate industrial requirements and environmental considerations. From a mechanistic view, as long as the shape and structure of a magnetic catalyst are maintained, it means that it will display high structural strength in successive recovery cycles and is not degraded.^247^

For instance, Boruah *et al.* introduced an ammonia-modified graphene sheet (AG) with Fe_3_O_4_ MNPs decorated on its surface. This magnetic catalyst with the synergistic effect caused by amide-functionalized graphene and Fe_3_O_4_ MNPs hamper the rate of electron–hole pair recombination, leading to recyclability for ten cycles, representing the high structural stability of the AG/Fe_3_O_4_ photocatalyst. Besides its enhanced stability and convenient separation, the high photocatalytic yield of this magnetic catalyst shows its potential for scaled-up applications.^248^

Fe_3_O_4_ anchored on reduced graphene oxide (rGO) through urushiol facile cross-linking (Fe_3_O_4_–U-rGO) exhibited a recycle stability of up to seven continuous cycles. One of the impressive factors affecting the structural stability is the urushiol molecule, which creates robust coordination to metal oxides and connects other materials *via* its structural phenolic hydroxyl groups. Due to the enhanced Fenton reactions of the Fe_3_O_4_–U-rGO magnetic composite, the generation of iron sludge and undesirable decomposition of H_2_O_2_ to H_2_O and O_2_ were inhibited.^249^

Also, for acceptable catalytic performance, metal leaching as a factor affecting the stability of catalysts under harsh conditions such as acidic medium should be considered. The coherence, correlation, and overall physical properties of the structure should be maintained.^250^ To investigate the catalyst activation after recycling cycles, comparing the adsorptions bands in the FT-IR spectrum of the recovered catalyst and the original one seems necessary.^251^ In this case, another work applied a heterogeneous magnetic catalyst, *i.e.*, an immobilized polymeric sulfonated ionic liquid on core–shell-structured Fe_3_O_4_/SiO_2_ composite (Fe_3_O_4_/SiO_2_-PIL), where the conversion of oil did not decrease significantly after five cycles of reutilizing, displaying the favorable recyclability of the catalyst. The durable structural stability of the catalyst indicated a strong attachment between the active acidic species and Fe_3_O_4_/SiO_2_ support. The reusability results verified a desirable heterogeneous catalyst from economic and environmental aspects compared to homogeneous catalysts, highlighting its applicability in the industrial production of biodiesel.^252^

It can be claimed that a magnetic catalyst is chemically stable when the deconjugation and chemical structure alteration of its active sites do not happen. In the case of metal oxide-based catalysts, sonication is proposed as an efficient way to recover magnetic catalysts given that agglomeration may occur during the catalytic procedure. The combination of magnetite NPs, *i.e.*, Fe_3_O_4_, and metal oxide NPs such as ZnO with a high agglomeration propensity can enhance the efficiency of the catalytic system through the adsorption of the target contaminants. The enhancement caused by magnetization correlates with the reduction in metal oxide agglomeration and catalytic performance improvement by the Fe^2+^ active octahedral sites of Fe_3_O_4_ reacting with H_2_O_2_ molecules, resulting in the formation of ˙OH and HO_2_˙ radicals, which eventually enhances the reusability of sonocatalysts and metal oxides.^250^ Besides, in ultrasound-assisted catalysis systems denoted as sonocatalysis, the activation of the sonocatalyst occurs under ultrasound irradiation, which accelerates the degradation rate *via* the production of extra reactive species. In this respect, many sonocatalysts, namely ZnO, TiO_2_–NiO, and TiO_2_, have been activated by ultrasound irradiation.^253–255^

## 7. Structural stability of micro and nanoscale magnetic catalytic systems

Stability is one of the critical parameters affecting the performance of catalysts. Different factors can affect their stability, such as pH,^256^ temperature,^239^ and reaction conditions. As the temperature increases, the dye removal rate increases. Peroxymonosulfate catalyzed by reusable graphite felt/ferriferous oxide dissociated into sulfate and hydroxyl radicals *via* thermolytic division of its O–O bond.

Additionally, a higher temperature produces more free radicals.^239^ Metal ion leaching is one reason that causes catalyst deactivation. In this case, the catalyst is immersed in aqueous solutions for a certain number of days. Subsequently, the leached metal concentration demonstrates the stability of the catalyst.^257^ Besides, the agglomeration of NPs diminishes the catalytic activity and stability.^243^ Magnetic nanoscale zero-valent iron (nZVI)@Ti_3_C_2_-based MXene nanosheets exhibited enhanced catalytic reactivity and stability due to its synergistic effect. Specifically, the Ti_3_C_2_-based MXene prevents the agglomeration of the nZVI particles (nZVIPs) and enhances the electron transition between the magnetic particles with a diameter of 10–40 nm.^245^

**Fig. 41:**
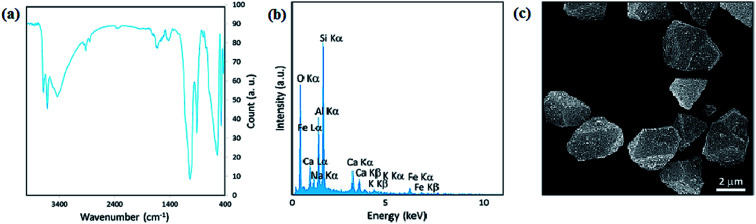
(a) FT-IR spectrum, (b) EDX spectrum, and (c) SEM image of the recovered pumice microparticles after six recycling cycles. This figure was adapted with permission from *Research on Chemical Intermediates*, 2020, **46**, 4113–4128.^244^

Different analyses are used to explore the structural stability of catalysts. Fig. 41a–c show the FT-IR, EDS, and SEM analyses of recovered magnetic pumice catalysts after six successive recycling cycles. The peaks at 580, 950, 1000, and 3390 cm^−1^ correspond to the characteristic peaks of the catalyst. Two new peaks emerged at 3600 cm^−1^, which are related to the ALO–H bonds in the internal alumina network and prove the presence of voids in pumice after complete rinsing. The EDS analysis did not display any changes in the structural elements of the recovered catalyst. Eventually, according to the SEM images, the structure, morphology, dispersion, and uniformity of the heterogeneous catalyst remained unchanged even after six recycling cycles.^244^

## 8. Turnover number (TON) and turnover frequency (TOF)

The turnover frequency (TOF) first widened into the scope of heterogeneous catalysis according to Boudart's definition, as follows: “Certainly, the catalytic activity, must be assigned to the exposed number of surface atoms of a determined kind. Thus, to put catalytic activity in words, it is a turnover number equal to the number of reactant molecules converted per minute per catalytic site for specified reaction conditions”.^264^ Also, the authentic IUPAC Gold Book defines the turnover frequency term as: “Commonly called the turnover number, N, and determined, as in enzyme catalysis, as molecules that react per active site in unit time”.^265^ TOF, turnover frequency (TOF = (yield/time)/amount of catalyst (mol)). TON, turnover number (TON = yield/amount of catalyst (mol)).^266^ Alternatively, to evaluate intrinsic properties such as catalytic efficiency and recycling factors, TOF and TON are two significant parameters. The TON is determined as the number of molecules that experience transformation by ratio of active sites to products in the presence of 1 g catalyst. Also, the TOF is calculated as TON/degradation time.^267^ Numerous methods that mainly utilize homogeneous metal complexes and heterogeneous systems for the hydration of nitriles have been reported. Alumina, potassium fluoride-doped Al_2_O_3_ and phosphates, silica-supported manganese oxides, modified hydroxyapatite, and ruthenium hydroxide coated on alumina and ferrites are examples of heterogeneous systems.

Nonetheless, due to their deficiency, laboriousness product and catalyst separation, requirement of inert atmosphere to conduct reactions for air-sensitive metal catalysts, poor catalyst reusability, very low TONs were obtained.^268^ In hydrogenation reactions of alkynes or alkenes, catalysts such as noble metals NPs represent high turnover numbers (TONs).^269^ Hamishehkar *et al.* synthesized glutathione-decorated gold-magnetic NPs (GSH-AuMNPs) as a competent recyclable catalyst. To express intrinsic activity, the TOF was concluded to be 12.5 h^−1^. This catalyst with high catalytic activity is a good candidate for biomedical and pharmaceutical applications.^270^ Koukabi *et al.* designed an air- and moisture-stable poly(2-acrylamido-2-methyl-1-propane sulfonic acid)-stabilized magnetic palladium catalyst with a core–shell structure. This catalyst was applied in Suzuki–Miyaura and Mizoroki–Heck reactions to produce coupling products with TON and TOF values of 14 143 and 4900 and 28 296 and 7424, respectively.^271^

## 9. Multifunctional micro and nanoscale magnetic catalytic systems

A wide variety of multifunctional systems based on magnetic nanoparticles (MNPs) and derivative nanocomposites has amazed scientists because of their manifold applications using one material. These materials are used in various fields, such as sensing, adsorption, medicine, energy, and electronics. Recently, Yang *et al.* synthesized a 3D coral-like layered porous magnetic eggshell membrane (ESM)/CuFe_2_O_4_ nanocomposite through a green and affordable route from useless materials, exhibiting multifunctional applications including adsorption, catalysis, and antibacterial properties in water treatment against Gram-positive bacteria *Staphylococcus aureus* (*S. aureus*) and Gram-negative bacteria *Escherichia coli* (*E. coli*). The maximum adsorption capacity of Congo red (CR) was evaluated to be 199.23 mg g^−1^. Further, the catalytic activity of 4-nitrophenol reduction using this magnetic nanocomposite was high, and the attained degradation rate was 98.9% in 5.5 min.^272^

Besides, multifunctional magnetic NiFe_2_O_4_@TiO_2_/Pt nanocomposites were prepared through the sol–gel procedure. In this work, the high performance of rapid photo-degradation of two azo dyes as water pollutants under UV-vis light, together with antibacterial activity against *Escherichia coli* (*E. coli*) bacteria were demonstrated.^273^ Kayani *et al.* worked on the sol–gel synthesis of magnetic ZnO NPs with different Fe dopant concentrations, ranging from 1–17%. Their studies confirmed the ferromagnetic behaviour of all the Fe-doped NPs. The highest antibacterial performance against *Escherichia coli* (*E. coli*) and *Pseudomonas aeruginosa* (*P. aeruginosa*) was acquired at 14–17% Fe doping content. The 1% Fe-doped ZnO photocatalyst revealed superior methylene blue (MB) degradation under sunlight.^274^ Ensafi *et al.* synthesized a magnetic spinel Fe_2_CuO_4_/rGO nanocomposite as a catalyst and adsorbent. The pyrolytic products resulting from the catalytic pyrolysis of discarded tires were liquid (pyrolytic fuel), gas (combustion gas), and char (activated carbon *via* gasification procedure). These products confirm the generation of valuable materials from waste. This nanocomposite acted as an adsorbent for mercury(ii) elimination from sewage, which corresponded to the Langmuir isotherm with a maximum adsorption capacity of 1250 mg g^−1^.^275^

Another study focused on an approach for the one-pot synthesis of a CoNi alloy-reduced graphene oxide ((CoNi_D_)_60_RGO_40_) multifunctional nanocomposite, which acted as a remarkable catalyst for the 4-nitrophenol reduction reaction with its *k*_app_ value determined to be 20.55 × 10^−3^ s^−1^ and 80–93% yield in the Knoevenagel condensation reaction. The easy separation of this magnetic nanocomposite and perfect performance of the recycled catalyst were noticeable. Also, this snowflake-like dendritic nanocomposite operated as an active electrode in supercapacitors. The high performance of this supercapacitor was exhibited by its specific capacitance of 501 F g^−1^ (at 6 A g^−1^) and energy density of 21.08 W h kg^−1^ at a power density of 1650 W kg^−1^. The synthesis of the snowflake-like dendritic (CoNi_D_)_60_RGO_40_ nanocomposite and its catalytic and supercapacitor applications were presented in this study.^276^

**Fig. 42:**
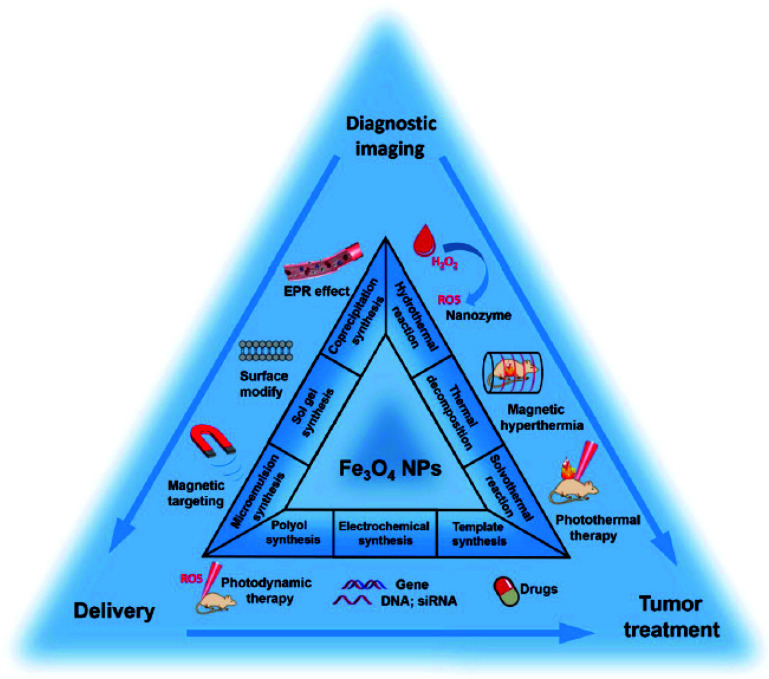
Scheme showing the synthesis of magnetic Fe_3_O_4_ NPs and their cancer diagnosis and healing applications. This figure was adapted with permission from *Theranostics*, 2020, **10**, 6278.^277^

Multifunctional and stimuli-responsive MNP- and derivative nanocomposite-based systems have emerged from nanobiotechnology with significant impacts on drug delivery, cancer diagnosis and treatment. These systems overcome the limitations of conventional therapeutic methods for cancer in recent decades. Among them, Fe_3_O_4_ MNPs have attracted attention from scientists due to their innate magnetic resonance imaging (MRI), drug delivery, biocatalytic activity, magnetic hyperthermia treatment (MHT), and stimuli-responsive therapy for multimodal approaches. The size and morphology are factors that determine the properties of these MNPs. There are three main routes presented for the synthesis of Fe_3_O_4_ MNPs, including physical, biosynthetic, and chemical approaches. Physical synthesis is a method that cannot entirely direct the size to the nano-scale. The biosynthetic process faces shortcomings of long synthesis time, low yield reaction, and broad size distribution. Thus, to overcome these disadvantages, chemical synthesis tends to be employed with superiorities such as facile procedure, low cost, high yield, and monodispersed MNPs. The various applications of Fe_3_O_4_ MNPs and their chemical synthetic strategies are presented in Fig. 42.^277^

**Fig. 43:**
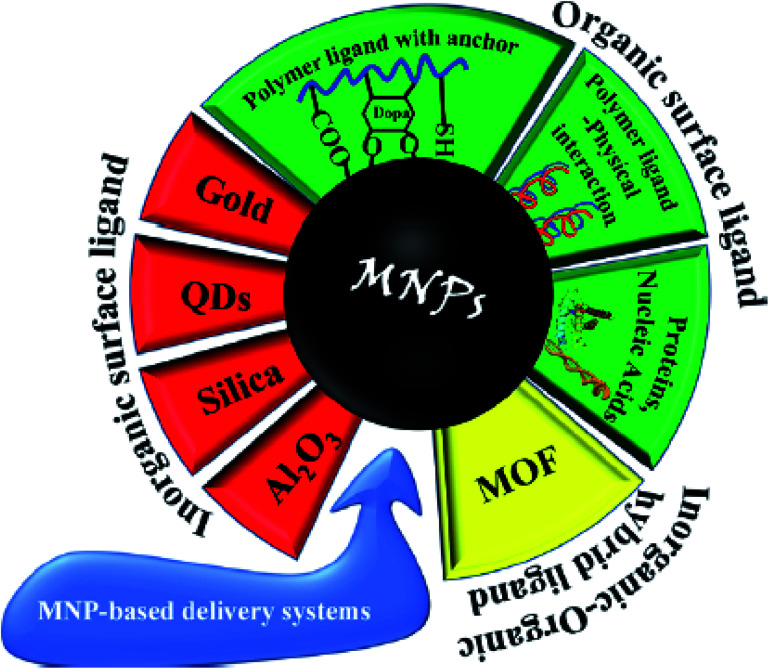
Illustration of MNP-based delivery systems and their versatile surface ligands (including inorganic surface ligands, organic surface ligands, and inorganic–organic hybrid ligands). This figure was adapted with permission from *Journal of Physics and Chemistry of Solids*, 2021, **148**, 109661.^278^

To better interact with biological media, surface-modified Fe_3_O_4_ MNPs with good biocompatibility, high stability, and stimuli responsiveness are applied in magnetic nanoscale systems. Ligand substitution and encapsulation are strategic processes. An interchange between the intrinsic surface hydrophobic ligands of MNPs and hydrophilic ligands take place to aid in their dispersity in the biological environment. However, although this process retains the initial hydrodynamic size of the MNPs, it causes metal atom leakage and surface flaws. Thus, the encapsulation/self-assembly process is employed to protect MNPs and to maintain their physical and chemical characteristics. The different functionalizing agents and surface ligands are depicted in Fig. 43.^278^ Feng *et al.* introduced poly(lactic-*co*-glycolic acid) (PLGA) drug-loaded magnetic Janus particles (DMJPs) as a multifunctional nanoscale system. In these three-sectioned Janus particles, Fe_3_O_4_ MNPs effectively released paclitaxel anticancer drugs with the assistance of an external magnetic field.^279^

Another theranostic nanosystem is multifunctional gold MNPs. According to their nanoscale and inimitable physicochemical properties, they display thriving multimodal imaging and cancer treatment applications. Also, from the biosafety perspective of Au MNPs, several types of studies proved their low toxicity in biomedical applications.^280^

**Fig. 44:**
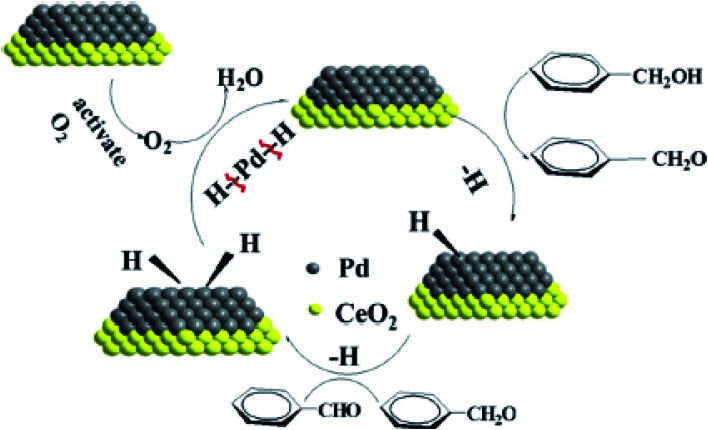
Mechanism of the green oxidation method for benzyl alcohol with Pd/Fe_3_O_4_@mCeO_2_ yolk–shell microsphere catalyst. This figure was adapted with permission from *Catalysis Letters*, 2016, **146**, 1321–1330.^283^

Multifunctional catalytic systems are supplementary systems that improve the catalytic activity through the interaction of more than one catalyst functionality.^281^ A chitosan/ionic liquid multifunctional catalyst was prepared with high catalytic activity and selectivity for the synthesis of dimethyl carbonates (DMC) due to its nucleophilic sites such as hydroxyl and amine groups and also absorption sites for CO_2_.^282^ Additionally, magnetic multifunctional catalytic systems are beneficial due to their ease of separation using a permanent external magnet to recycle the catalysts. According to the desirable conversion and selectivity of multifunctional catalysts applied in the green oxidation method of benzyl alcohol, Ye *et al.* introduced Pd/Fe_3_O_4_@mCeO_2_ yolk–shell microspheres, as presented in Fig. 44. The catalyst exhibited a good performance of 80.5% conversion of benzyl alcohol and benzaldehyde selectivity of 94.8%.^283^

In another study, NiFe–NiFe_2_O_4_ was synthesized *via* the solution blow spinning (SBS) method, which exhibited good electrocatalytic activity in the oxygen evolution reaction (OER) with a noteworthy turnover frequency (TOF) of 4.03 s^−1^.^284^ Another electrocatalyst for the OER and also water splitting, resulting in the production of pure H_2_, is cobalt ferrite (CoFe_2_O_4_) powder obtained using agar–agar from Rhodophyta. The TOF of this electrocatalyst was 8.8 × 10^−2^ s^−1^.^285^ Also, Nagashri *et al.* prepared a cost-effective copper(ii) complex with a 1,10-phenanthroline derivative catalyst for H_2_ evolution as a fuel with a TON and TOF of 15 600 and 8100, respectively.^286^ Moreover, multifunctional catalytic systems present a satisfactory performance in the Suzuki–Miyaura and Mizoroki–Heck cross-coupling reactions. In this regard, the oxygen insensitive and phosphine-free MNP/HPG–CA/Pd catalyst with a turnover frequency of 372 h^−1^ was effortlessly recovered and reused many times.^287^ Similarly, a one-pot hydrothermal-synthesized Pd–Fe_3_O_4_/rGO nanocomposite with a TOF of up to 1449 h^−1^ was applied in C–C coupling reactions.^288^

Magnetic core–shell-structured multifunctional heterogeneous catalysts exhibit synergistic effects from each constituent, such as preferable magnetic properties, catalytic activity, and structural stability. A layer-by-layer assembled Fe_3_O_4_@PDA-Pd@[Cu_3_(BTC)_2_] nanocomposite was employed for the reduction of 4-nitrophenol and Suzuki–Miyaura cross-coupling reactions.^77^Table 5 summarizes the information of some magnetic catalytic systems that exhibit versatility in chemical reactions.

**Table 5:** Brief information of some other multifunctional catalytic systems

Entry	Catalyst	Active site	Main function	TON and TOF	Ref.
1	FeS_2_/CoS_2_ nanosheets	“S” vacancies	Electrocatalyst	TOF = 0.446 (s^−1^)	289
2	Co@MOF	Co	Catalyst	TOF = 0.8 (h^−1^)	290
3	Au/MOF-199	Au, Cu content in MOF	Catalyst	TON = 363, TOF = 738 (h^−1^)	291
4	Ru(ii)–PNN	Ru	Catalyst	TON = 4400, TOF = 2500 (h^−1^)	292
5	[κ^4^-Tptm]ZnOSiMe_3_	Zn	Catalyst	TON = 10^5^, TOF = 1.6 × 106 (h^−1^)	293
6	H_3_PW_12_O_40_	Pt	Catalyst	TOF = 5.2 (h^−1^)	294
7	C_18_H_26_IrN_3_O_7_S·3.5H_2_O	OH	Catalyst	TON = 7280, TOF = 2600 (h^−1^)	295
8	Al/SiO_2_	Al	Ethanol dehydration and *m*-xylene isomerization	TOF = 5.0 (×10^−4^ s^−1^ per site)	296
9	FeNi_3_N/NG	Ni, pyridinic N graphitic N, Fe	Electrocatalyst	TOF = 1.0 (s^−1^)	297
10	MoS_2_@NF	Mo	Catalyst	TOF = 2.54H_2_ s^−1^	298

## 10. Conclusion and future outlook

In this survey, a brief overview of the recent advancements in magnetic micro- and nanoscale catalytic systems was presented. Concurrently, their myriad of benefits and convenience, such as facile recovery and high chemical and structural stability, in the degradation and synthesis of assorted pharmaceutical compounds was discussed. Herein, the structure and properties of magnetic catalytic systems were classified, and all the recent accomplishments by researchers were briefly implied in this context. Furthermore, the synergistic effects between organic and inorganic catalysts as hybrid micro- and nanoscale magnetic composites were considered. In general, magnetic catalytic systems are developing rapidly. In this regard, researchers are trying to optimize previous processes both in terms of cost and time, as well as in terms of improving catalytic structures. Alternatively, it is imperative to focus on environmentally friendly structures and extracted materials from nature. These materials do not harm the environment, and are considered an opportunity in terms of mass production and industrial use.

As a path to the future, attention to porous structures and frameworks is a priority. Due to their high porosity, these structures can act as reaction reactors and accelerate catalytic processes. These structures include metal–organic frameworks (MOFs) as well as a newer category, covalent organic frameworks (COFs). In addition to high porosity, COFs can also host magnetic nanoparticles, which aid the convenient separation of catalysts. Moreover, the prominent features of these frameworks are their lower density than MOFs and their high stability under acidic, alkaline, and redox conditions. Besides, attention should be given to magnetic heterostructures, which possess features beyond their single components, resulting in various applications in biomedicine and catalyst scopes. The insight into the reaction parameters, including ligands and counter anions, leads to the application of magnetic heterostructures in the desired fields. Regarding the biomedical application of magnetic heterostructures, the requirement of their long-term appraisal in terms of biotoxicity and metabolism of the magnetic heterostructures seems vital, while in terms of catalytic applications, stability enhancement has been highlighted as the major issue.^299^

Alternatively, the preparation approach of metal complexes containing immobilized MNPs enables phase tunability of the magnetic oxides applying a single path (Fe_3_O_4_, γ-Fe_2_O_3_, and α-Fe_2_O_3_). Even though the immobilization of MNPs on metal complexes results in higher activity and selectivity in the catalytic systems, the hydrophobicity may affect their catalytic performance. However, silica- and carbon-coated MNPs can prevent the aggregation of MNPs, providing functional groups to immobilize their active catalytic sites.^300^ Magnetic biochar is another magnetic catalytic system originating from a wide range of sources, which can be prepared *via* various routes and employed in the catalytic degradation of dyes, antibiotics, herbicides, and other organic contaminants. Although MBC catalysts exhibit the benefits of excellent stability, good reusability, and acceptable organic pollutant degradation yield, insight into many related facets to the mechanism of MBC is scarce. For instance, more environmental considerations in the case of metal leaching of some metal-loaded MBCs, profound studies on the routes for the regeneration of MBC catalysts, and concise research on the incomplete mineralization of degraded contaminants should be undertaken.^38^ Finally, the use of hybrid composites also requires more attention due to the catalytic benefits of both organic and inorganic compounds.

Table 6 lists the abbreviations used herein and their full definitions.

**Table 6:** The abbreviations used herein and their related definitions

Abbreviation	Definition
MNPs	Magnetic nanoparticles
MCRs	Multicomponent reactions
MOF	Metal–organic framework
COF	Covalent–organic framework
MCSs	Magnetic catalytic systems
PAHs	Polycyclic aromatic hydrocarbons
CIP	Ciprofloxacin
TOF	Turnover frequency
TON	Turnover number
DMC	Dimethyl carbonates
SBS	Solution blow spinning
OER	Oxygen evolution reaction
GA	Gallic acid
PLGA	Poly(lactic-*co*-glycolic acid)
MMT	Montmorillonite
GO	Graphene oxide
PDA	*Ortho*-phenylenediamine
nZVI	Nanoscale zero-valent iron
TMB	3,3,5,5-Tetramethylbenzidine
AA	Ascorbic acid
CR	Congo red
MB	Methylene blue
GSPE	Graphitic screen-printed electrode
VPMP	Volcanic pumice magnetic particles
DEA	Diethanolamine
ESM	Eggshell membrane
MHT	Magnetic hyperthermia treatment
DMJP	Drug-loaded magnetic Janus particles
ICP-AES	Inductively coupled plasma atomic emission spectroscopy
MRI	Magnetic resonance imaging
MCM	Microwave combustion method
CCM	Conventional combustion method
CMC	Carboxymethylcellulose

## Conflicts of interest

There are no conflicts of interest to declare.

## Supplementary Material
